# Characterization and Analysis of the Full-Length Transcriptomes of Multiple Organs in *Pseudotaxus chienii* (W.C.Cheng) W.C.Cheng

**DOI:** 10.3390/ijms21124305

**Published:** 2020-06-17

**Authors:** Li Liu, Zhen Wang, Yingjuan Su, Ting Wang

**Affiliations:** 1 School of Life Sciences, Sun Yat-sen University, Guangzhou 510275, China; liuli11307@163.com (L.L.); zhenwangzhenwang@163.com (Z.W.); 2 Research Institute of Sun Yat-sen University in Shenzhen, Shenzhen 518057, China; 3 College of Life Sciences, South China Agricultural University, Guangzhou 510642, China

**Keywords:** *Pseudotaxus chienii*, PacBio Iso-Seq, transcriptome, multiple organs, phenylpropanoid biosynthesis pathway, plant–pathogen interactions

## Abstract

*Pseudotaxus chienii*, a rare tertiary relict species with economic and ecological value, is a representative of the monotypic genus *Pseudotaxus* that is endemic to China. *P. chienii* can adapt well to habitat isolation and ecological heterogeneity under a variety of climate and soil conditions, and is able to survive in harsh environments. However, little is known about the molecular and genetic resources of this long-lived conifer. Herein, we sequenced the transcriptomes of four organs of *P. chienii* using the PacBio Isoform Sequencing and Illumina RNA Sequencing platforms. Based on the PacBio Iso-Seq data, we obtained 44,896, 58,082, 50,485, and 67,638 full-length unigenes from the root, stem, leaf, and strobilus, respectively, with a mean length of 2692 bp, and a mean N50 length of 3010.75 bp. We then comprehensively annotated these unigenes. The number of organ-specific expressed unigenes ranged from 4393 in leaf to 9124 in strobilus, suggesting their special roles in physiological processes, organ development, and adaptability in the different four organs. A total of 16,562 differentially expressed genes (DEGs) were identified among the four organs and clustered into six subclusters. The gene families related to biotic/abiotic factors, including the TPS, CYP450, and HSP families, were characterized. The expression levels of most DEGs in the phenylpropanoid biosynthesis pathway and plant–pathogen interactions were higher in the root than in the three other organs, suggesting that root constitutes the main organ of defensive compound synthesis and accumulation and has a stronger ability to respond to stress. The sequences were analyzed to predict transcription factors, long non-coding RNAs, and alternative splicing events. The expression levels of most DEGs of C2H2, C3H, bHLH, and bZIP families in the root and stem were higher than those in the leaf and strobilus, indicating that these TFs may play a crucial role in the survival of the root and stem. These results comprise the first comprehensive gene expression profiles obtained for different organs of *P. chienii*. Our findings will facilitate further studies on the functional genomics, adaptive evolution, and phylogeny of *P. chienii*, and lay the foundation for the development of conservation strategies for this endangered conifer.

## 1. Introduction

*Pseudotaxus chienii* (Taxaceae), a rare tertiary relict species, is a representative of the monotypic genus *Pseudotaxus* that is endemic to China [1]. The distinguishing feature of this species is the presence of a white aril and two distinct white stomatal bands on the underside of mature leaves [1]. This species is a dioecious evergreen woody shrub or tree that grows in subtropical mountain forests [2]. According to the International Union for Conservation of Nature (IUCN) Red List, *P. chienii* has been classified as a vulnerable species, due to the low conception rate of the female plants, its low seed germination rate, and its slow natural regeneration [3,4]. Populations of *P. chienii* are sparsely distributed in forest valleys or on cliffs, primarily growing in shallow and acidic soil or in the crevices of rocks [3,5]. *P. chienii* can adapt to habitat isolation and ecological heterogeneity well, in a variety of climate and soil conditions [5,6]. As an old rare species with a long life, long generation time, and long history, *P. chienii* is highly valued, and suitable for the investigation of adaptive evolution. Although a previous study on *P. chienii* focused on the use of second-generation sequencing technology to characterize the transcriptome [7], the majority of the studied unigenes were not full-length (FL) cDNA sequences, and did not yield sufficient gene expression profiles of the multiple organs. A few studies have focused on multiple organs in species, showing that organ differentiation is the main driver of transcriptome recombination [8]. In *Arabidopsis thaliana*, each organ type has a specific expression pattern, and the degree to which organs share expression profiles, is highly correlated with the biological relationship of the organ types [8]. Similar findings have been reported for conifers, such as white spruce (*Picea glauca*) [9], maritime pine (*Pinus pinaster*) [10], and Norway spruce (*Picea abies*) [11]. These findings suggest that the consideration of multiple organs, including the determination of organ-specific expression patterns, is a powerful approach to characterizing the transcriptomic complexity across a whole organism. The current work attempts to fill this gap by studying the multiple organ transcriptomes of *P. chienii*.

Conifer transcriptome studies are the molecular basis for our understanding of the plant growth, development, and stress adaptation. Conifers are one of the longest living plants on the earth, and have survived through numerous climate oscillations, pest infestations, and natural disasters that have occurred over time [12,13,14]. They have evolved many sophisticated mechanisms against various environmental stresses, most of which depend on changes in gene expression [15]. Several important candidate genes and metabolic pathways have been identified to respond to biotic and abiotic stresses in some conifer species. Hall et al. [16] used transcriptome assemblies to investigate the defense genes of six tissue/organ types from *Pinus banksiana* and *Pinus contorta*, and identified candidate full-length prenyl transferase, terpene synthase (TPS), and cytochrome P450 (CYP450) genes. So far, the majority of the TPSs and CYP450s found in plants have been confirmed to function in plant defense and stress resistance. Conifer trees produce a number of terpenoid metabolites, which act as a chemical barrier against insects and pathogens [17]. CYP450s can promote plant growth and protect plants from stresses through a variety of biosynthetic pathways, such as flavonoids, lignins, phenolics, antioxidants, and phenylpropanoids [18]. Other gene products are important for abiotic stresses (e.g., drought and low-temperature stresses), including dehydrin, endochitinase, heat shock protein (HSP), phenylalanine ammonia-lyase (PAL), and late-embryogenesis abundant (LEA) proteins [19,20]. HSPs, as molecular chaperones, can protect plants against stress by reconstructing normal protein conformation [21]. PAL is a key enzyme catalyzing the first step in the phenylpropanoid biosynthesis pathway, which is an important process for the synthesis of defensive compounds [22,23]. Several genes involved in the phenylpropanoid pathway produce physical and chemical barriers against stress, such as the formation of lignin and phenylpropanoid compounds [22]. The plant–pathogen interactions also may play a role in conifer defense, which is an important process that allows plants to avoid infection and achieve immunity [23]. However, despite the importance of these gene families and metabolic pathways in conifer species, their systematic and thorough analysis in *P. chienii* is still lacking.

Transcriptome analysis could identify the type and number of intracellular key genes and reveal the metabolic pathways at a molecular level [24,25]. At present, several technologies have been applied for transcriptome sequencing. Among these, second-generation sequencing technology (e.g., Illumina RNA Sequencing (Illumina RNA-Seq)) can characterize gene expression levels at a greater sequencing depth [26]. With the advancement of Illumina RNA-Seq technology and low sequencing costs, we have a good opportunity to uncover the transcriptomic sequences of individual species. However, the lack of reference genome sequences for some species makes the assembly and annotation of the transcriptome incomplete and error-prone. Furthermore, it is difficult to identify FL transcripts based on the short reads generated by RNA-Seq. With the development of sequencing technology, third-generation sequencing technology (e.g., PacBio Isoform Sequencing (PacBio Iso-Seq)) can now be used to sequence FL or near FL transcripts without assembly, which helps overcome the limitations of second-generation sequencing technology. This technique has the advantages of long read lengths, high accuracy, high sensitivity, and a low degree of bias [27]. To date, PacBio Iso-Seq has been utilized to identify alternative splicing (AS) events, and also a large number of FL transcripts from many species lacking reference genome sequence information [28].

In this study, we combined the PacBio Iso-Seq and Illumina RNA-Seq technologies to reliably perform comprehensive transcriptome analyses and characterize the gene expression profiles in four organs of *P. chienii*. The aims of our study include: (i) generating reference transcriptome sequences for *P. chienii* from four organ types by using the PacBio Iso-Seq technique; (ii) detecting transcription factors (TFs), long non-coding RNAs (lncRNAs), and AS events; (iii) exploring gene expression patterns and differentially expressed genes (DEGs) among the four organs; (iv) exploring organ-specific unigene expression profiles; and (v) identifying candidate genes and metabolic pathways for adaptation to biotic and abiotic factors. This study will increase our understanding of the FL transcriptome complexity of *P. chienii* and provide a valuable molecular-level reference for future studies on the functional genomics, adaptive evolution, phylogeny, and conservation of *P. chienii* and other conifer species.

## 2. Results

### 2.1. The Full-Length Sequences of PacBio Iso-Seq

**Figure 1:**
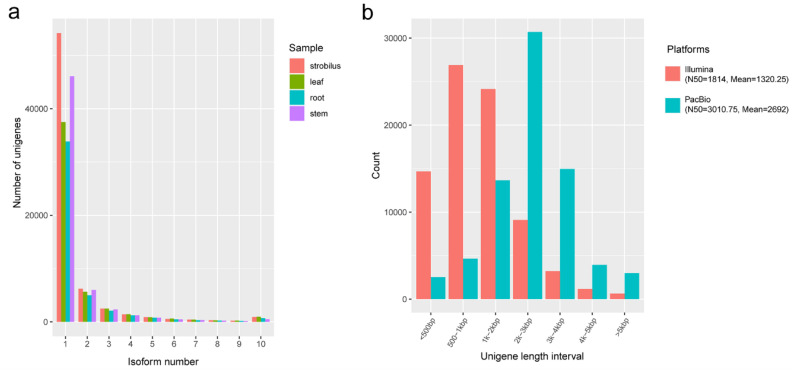
(**a**) Distribution of isoform numbers for unigenes in four organs of *P. chienii*; (**b**) comparison of unigene length distribution using different sequencing platforms.

Based on PacBio Iso-Seq, 3,589,223, 4,242,683, 4,905,002, and 5,427,724 subreads representing 8.21, 8.35, 11.42, and 11.22 Gbp were generated for the root, stem, leaf, and strobilus, with a mean length of 2289, 1968, 2328, and 2068 bp, respectively (Table S1). Circular consensus sequences (CCSs) were obtained from the subreads by self-correction (Figure S1). In total, 223,184, 212,703, 325,323, and 331,286 CCSs were obtained for the root, stem, leaf, and strobilus, respectively. By detecting the sequences, 174,922, 175,321, 270,445, and 269,571 were identified as full-length non-chimeric (FLNC) reads for the root, stem, leaf, and strobilus, with a mean length of 2537 bp and a mean N50 length of 2773 bp. Based on the iterative clustering for error correction (ICE) algorithm and the polishing of the Arrow algorithm, 80,434, 92,515, 142,159, and 126,538 high-quality (HQ), FL, and polished consensus isoforms were generated for the root, stem, leaf, and strobilus, respectively (Table S1). The polished consensus isoforms were further corrected using Illumina RNA-Seq data (Table S3) to improve the data quality. Finally, after removing the redundant sequences using CD-HIT, 44,896, 58,082, 50,485, and 67,638 unigenes were obtained for the root, stem, leaf, and strobilus, respectively, with a mean length of 2692 bp and mean N50 length of 3010.75 bp (Table S1). Most unigenes had a length ranging from 2000 to 4000 bp, accounting for 62.90% of the total number (Figure 1b). Most of the unigenes were single-isoform genes present in all four organs (Figure 1a). Based on Benchmarking Universal Single-Copy Ortholog (BUSCO) analyses, approximately 76.88% of the 1440 expected embryophyte genes were identified as complete, suggesting that the integrity of the *P. chienii* transcriptome was high (Figure S2).

A total of 197,174 reference transcript sequences for *P. chienii* were generated from four organs using PacBio Iso-Seq, with a 529.06 Mbp total nucleotide bases. The mean length of all transcripts and the mean N50 length were 2683 bp and 3024 bp, respectively (Table S2). A total of 288 mRNAs derived from *P. chienii* were reported in the National Center for Biotechnology Information (NCBI) database. All 197,174 FL transcripts were aligned to 288 mRNAs derived from the NCBI database using BLASTN, and the results revealed 287 (99.65%) previously reported mRNAs with similarities to our FL transcripts, with sequence identities ranging from 96.89% to 100% (*E*-value < 1.0 × 10^−10^). Among them, 267 previously reported mRNAs had a sequence identity of 99% or more, which suggests that the PacBio FL transcript database we generated for *P. chienii* has good accuracy.

### 2.2. De Novo Assembly of Illumina RNA-Seq Data

Based on Illumina RNA-Seq, approximately 215 million raw reads were produced from four organs of *P. chienii*. After filtering, for the root, stem, leaf, and strobilus, 51.07, 56.42, 61.11, and 41.45 million clean reads representing 7.66, 8.46, 9.17, and 6.22 Gbp were obtained, with a Q20 of 97.16%, 98.03%, 96.9%, and 97.28%, respectively (Table S3). Based on these clean reads, 66,126, 79,842, 52,207, and 60,391 unigenes, respectively, for the root, stem, leaf, and strobilus were assembled de novo, with a mean N50 of 1814 bp, and a mean length of 1320.25 bp (Table S4). Most of the unigenes (80.70%) from Illumina RNA-Seq had a length of less than 2000 bp, and only 2332 (0.90%) were more than 5000 bp (Table S5). The comparison of the unigene length distribution for different sequencing platforms is shown in Table S5 and Figure 1b.

### 2.3. Functional Annotation

To derive the most informative and complete information for functional annotation of the unigenes derived from the four organs of *P. chienii*, we performed a similarity search using these sequences in seven public databases, including the NCBI non-redundant protein (Nr), NCBI non-redundant nucleotide (Nt), Swiss-Prot, Pfam protein families, NCBI euKaryotic Ortholog Groups (KOG), Gene Ontology (GO), and Kyoto Encyclopedia of Genes and Genomes (KEGG) databases. For the root, stem, leaf, and strobilus, 40,166 (89.46%), 49,593 (85.38%), 47,697 (94.48%), and 58,654 (86.72%) unigenes were respectively annotated in at least one of the seven databases, corresponding to 13,610, 13,811, 15,489, and 16,641 unigenes being annotated in all databases (Table 1), indicating that our FL transcripts covered a large number of genes of *P. chienii*, and that most of these were likely functional.

**Figure 2:**
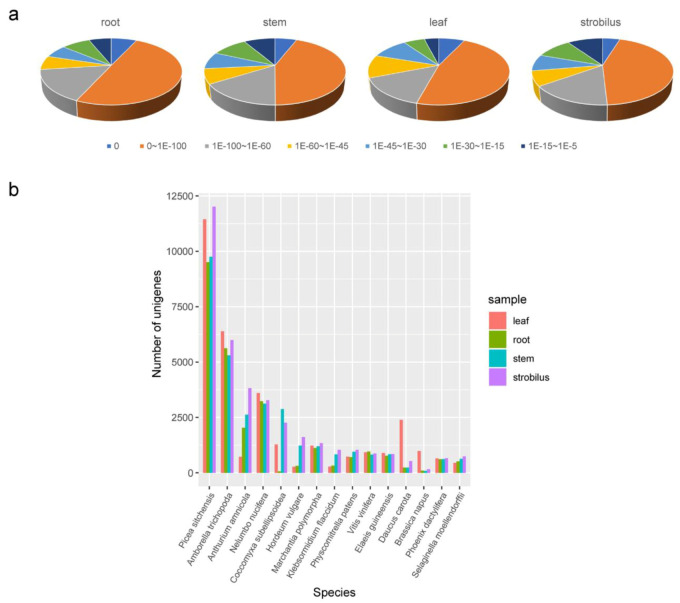
The distribution of homologous species annotated in the NCBI non-redundant protein (Nr) database. (**a**) The *E*-value distribution of the hit unigenes; (**b**) Top 10 hit species for unigenes identified in the four organs of *P. chienii*.

The sequences were compared to those of homologous species by aligning the unigenes using information in the Nr database. The top three homologous species in the root, stem, and leaf were all distributed in *Picea sitchensis* (9507, 9751, and 11,443 unigenes), *Amborella trichopoda* (5622, 5296, and 6392 unigenes), and *Nelumbo nucifera* (3231, 3124, and 3601 unigenes). In the strobilus, the top three homologous species were *P. sitchensis* (12,019 unigenes), *A. trichopoda* (5995 unigenes), and *Anthurium amnicola* (3825 unigenes) (Figure 2). Unsurprisingly, the top homologous species was a conifer. The *E*-value distribution showed that most of the unigenes shared higher homology with their hits from homologous species (*E*-value < 1 × 10^−100^), and that the distribution patterns of *E*-values were generally similar among the four organs (Figure 2a).

GO terms were used to functionally classify the *P. chienii* unigenes. For the root, stem, leaf, and strobilus, 27,425 (61.09%), 34,770 (59.86%), 30,132 (59.69%), and 40,467 (59.83%) unigenes, respectively, were assigned GO terms, which were classified into three main categories, including 17,898, 23,013, 19,977, and 27,076 unigenes from “biological process”; 23,813, 29,877, 25,958, and 34,346 unigenes from “molecular function”; and 8535, 11,567, 9398, and 13,173 unigenes from the “cellular component” (Table S6). For the biological process category, the major subcategories were “metabolic process” (GO: 0008152), “cellular process” (GO: 0009987), and “single-organism process” (GO: 0044699). In the molecular function category, the unigenes involved in “binding” (GO: 0005488), “catalytic activity” (GO: 0003824), and “transporter activity” (GO: 0005215) were highly represented. The major subcategories of cellular component were “cell” (GO: 0005623), “cell part” (GO: 0044464), and “membrane” (GO: 0016020) (Figure S3). These representative functions are the most fundamental biological functions necessary for cellular life, and are therefore abundant in all plants. The patterns of the GO annotations for these functions (at level 2) were generally similar across four organs of *P. chienii*. The category “response to stimulus” (GO: 0050896) is very important for the defense systems of conifer trees, and approximately 8.92% (2446), 8.53% (2967), 8.34% (2512), and 7.79% (3151) of the unigenes for root, stem, leaf, and strobilus were classified under this category (Table S6). In the GO term “response to stimulus”, “cellular response to stimulus” (GO: 0051716), “response to stress” (GO: 0006950), and “response to chemical” (GO: 0042221) were the top 3 annotations in the four organs at GO level 3.

We used the KEGG database to investigate the biological pathways. As with the GO functional classification, the percentages of different KEGG pathway classes were quite similar among the four organs of *P. chienii*. For the root, stem, leaf, and strobilus, 37,452 (83.42%), 43,929 (75.63%), 46,196 (91.50%), and 51,357 (75.93%) unigenes were assigned to 46 KEGG pathway classes, comprising 367, 374, 367, and 375 subclass pathways, respectively (Table S7). “Carbohydrate metabolism” and “signal transduction” were the top two pathways with the greatest number of unigenes in the four organs (Figure S4). For the root, stem, leaf, and strobilus, 1684, 2385, 2397, and 3502 unigenes were assigned to “carbohydrate metabolism”, and 2024, 2610, 2021, and 3264 unigenes were assigned to “signal transduction”. There was a higher number of unigenes associated with these two pathways in the strobilus than in the other three organs.

**Figure 3:**
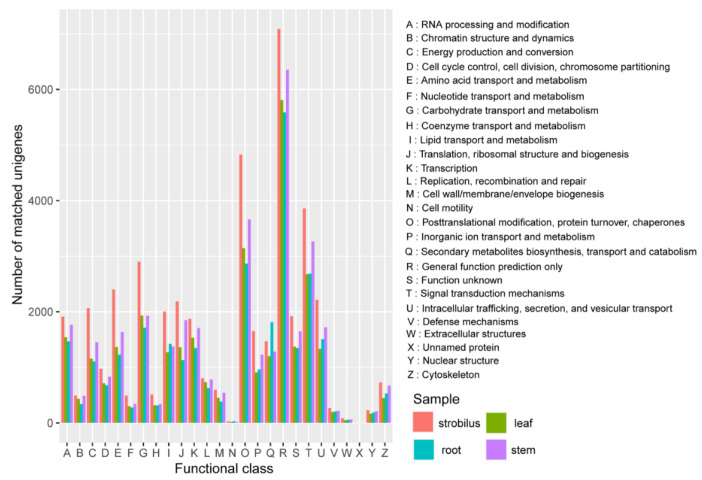
Eukaryotic Ortholog Group (KOG) classification of unigenes.

For more comprehensive annotation, all unigenes were searched against the KOG database. For the root, stem, leaf, and strobilus, 26,123 (58.19%), 31,452 (54.15%), 26,955 (53.39%), and 38,723 (57.25%) unigenes were classified into 26 KOG categories, respectively (Figure 3). Similar to the GO classification and KEGG pathways, the distribution patterns of KOG categories were generally similar among the four organs. The undefined functional “general function prediction only”, “posttranslational modification, protein turnover, chaperones”, and “signal transduction mechanisms” were the top three categories in all four organs. “Defense metabolites” is very important for the physiology and evolution of conifers, and 207, 216, 195, and 267 of the unigenes for the root, stem, leaf, and strobilus were clustered into this category.

### 2.4. Identification of CDSs, TFs, and LncRNAs

For the root, stem, leaf, and strobilus, we identified 45,020, 58,539, 50,842, and 68,851 putative coding sequences (CDSs) with a mean length of 1293.5 bp, and 26,881, 32,398, 29,235, and 39,633 carried complete CDSs. Most (80.93%, 78.80%, 78.88%, and 84.49% for root, stem, leaf, and strobilus) of these CDSs were shorter than 2000 bp, and only 2254, 3169, 2972, and 2075 (5.01%, 5.41%, 5.85%, and 3.01%) were longer than 3000 bp. There were 37,204, 45,390, 42,602, and 55,482 5′ untranslated regions (UTRs) and 43,643, 55,685, 49,110, and 66,243 3′ UTRs for the root, stem, leaf, and strobilus (Table S8).

**Figure 4:**
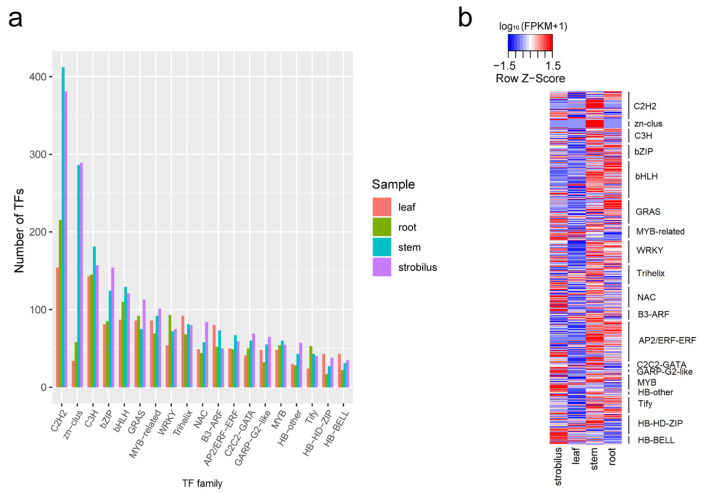
Identification of the transcription factors (TFs). (**a**) Identification of the top 15 TF families in four organs of *P. chienii*; (**b**) a heatmap of differentially expressed genes (DEGs) in the top 15 TF families. A scale indicates the color assigned to log_10_(FPKM + 1). Red indicates high expression, and blue indicates low expression.

For the root, stem, leaf, and strobilus, we identified 1678, 2432, 1706, and 2497 TFs from 66 different families, using the iTAK pipeline (Table S9). The top 15 TF families identified in the four organs are shown in Figure 4a. Among them, C2H2, C3H, bHLH, and bZIP were the most commonly represented TF families in all four organs of *P. chienii*. We found 215, 412, 154, and 381 putative C2H2 TFs in the root, stem, leaf, and strobilus, an obviously highest number than that for other TF families, followed by C3H (145, 181, 143, and 157), bZIP (85, 124, 81, and 154), and bHLH (110, 129, 87, and 121). We further identified the DEGs of the top 15 TF families among the four organs. We found that most DEGs of C2H2, C3H, bHLH, and bZIP families had higher expression levels in the root and stem than those in the leaf and strobilus (Figure 4b). The identification of numerous TFs provides abundant resources for the further research of specific TFs under the various life processes and environmental stresses of *P. chienii*.

We used four computational approaches (PLEK, CNCI, CPC, and Pfam) to identify lncRNAs with high confidence. We ultimately identified 2470, 3853, 1218, and 3091 unigenes (accounting for 5.50%, 6.63%, 2.41%, and 4.57% of the total unigenes, respectively) as putative lncRNAs in the root, stem, leaf, and strobilus, respectively. The number of lncRNAs in leaf was lower than that in the other three organs. The length of the lncRNAs varied from 200 to 9513 bp. Most (87.39%) of these lncRNAs were shorter than 3000 bp, and only 101 (0.96%) were longer than 5000 bp (Figure S5a). The mean length of the lncRNAs was 1382 bp, which was shorter than the mean length of all unigenes (2692 bp). The expression levels of differentially expressed lncRNAs are shown in a heatmap (Figure S5b). Although we identified many lncRNAs, the functions of these lncRNAs needs to be further characterized.

### 2.5. AS Analysis

**Figure 5:**
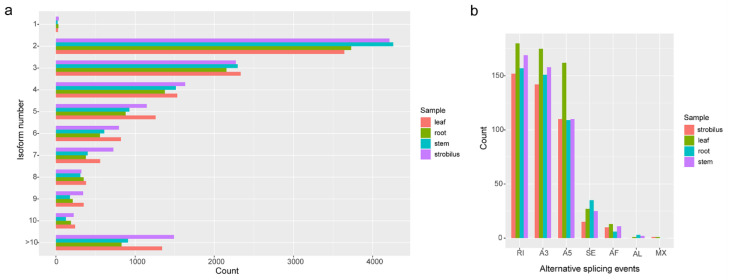
Identification of alternative splicing (AS) events. (**a**) The distribution of isoform numbers for UniTransModels in four organs; (**b**) the number of AS events in four organs.

For the root, stem, leaf, and strobilus, 10,681, 11,556, 12,485, and 13,206 unique transcript models (UniTransModels) were captured, of which 99.71%, 99.81%, 99.79%, and 99.73% had more than one isoform. In total, 11.30% and 10.73% of the UniTransModels had more than 10 isoforms in the strobilus and leaf, and these percentages were lower (7.87% and 7.75%) for the stem and root (Figure 5a). We identified 461, 475, 559, and 430 AS events in root, stem, leaf, and strobilus covering 3888, 4178, 4035, and 4113 UniTransModels, respectively. Retained introns (RIs) were identified as the predominant AS event in all four organs, and we identified 157, 169, 180, and 152 (accounting for 34.06%, 35.58%, 32.20%, and 35.35%) RIs in the root, stem, leaf, and strobilus, respectively. The types of alternative 3′ splice sites (A3) (151, 158, 175, and 142 for the root, stem, leaf, and strobilus) and alternative 5′ splice sites (A5) (109, 110, 162, and 110 for the root, stem, leaf, and strobilus) were followed. Mutually exclusive exons (MX) and alternative last exons (AL) were the least frequent (Figure 5b).

### 2.6. Gene Expression Quantification

The Illumina clean reads of each organ were mapped to the 197,174 FL reference transcript sequences, and the mapping rates were 85.89%, 83.97%, 89.56%, and 84.82% for the root, stem, leaf, and strobilus (Table S10), respectively, indicating ideal sequencing and mapping. The expression level of each unigene in each organ was analyzed by estimating the fragments per kilobase of transcript sequence per million base pairs sequenced (FPKM). FPKM interval analysis showed that the FPKM values between 0 and 0.1 accounts for 60% of all unigenes in four organs, followed by FPKM values between 0.1 and 1, accounting for approximately 16% of all unigenes; FPKM values were between 1 and 5 in about 11% of all unigenes; FPKM values were between 5 and 15 in about 4.5% of all ungenes; FPKM values were between 15 and 60 in about 3% of all unigenes; and FPKM values were greater than 60 in about 1% of all unigenes (Figure S6a). Boxplots of the FPKM values in the four organs are shown in Figure S6b. The average FPKMs of the root, stem, leaf, and strobilus were 4.32, 4.79, 5.23, and 5.06, respectively. The unigene expression levels for the root and stem were slightly lower than those for the leaf and strobilus.

### 2.7. DEGs and Functional Enrichment Analysis

**Figure 6:**
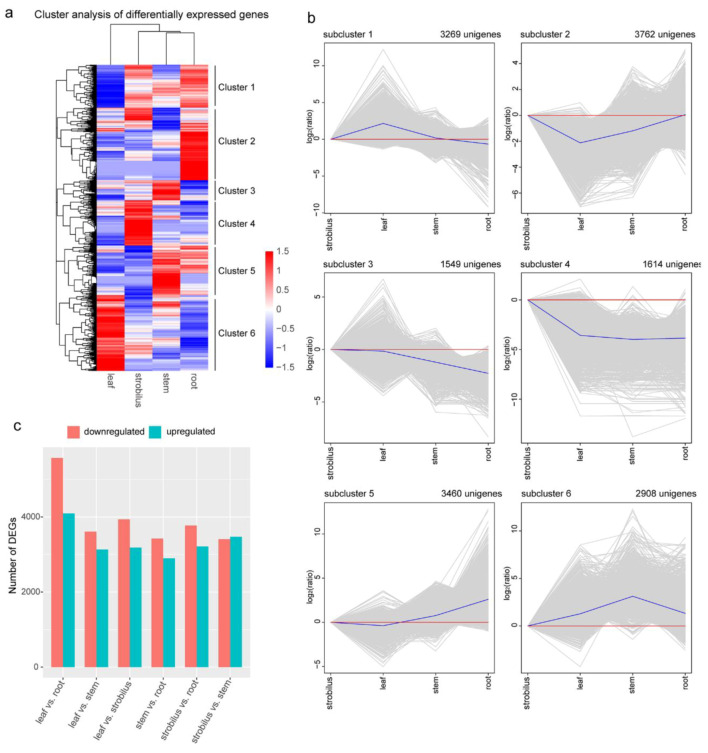
Differentially expressed genes (DEGs) among four organs of *P. chienii*. (**a**) A heatmap of the expression patterns of 16,562 DEGs. A scale indicates the color assigned to log_10_(FPKM + 1). Red indicates high expression, and blue indicates low expression. (**b**) The six subclusters of the 16,562 DEGs were clustered. The number of unigenes in each subcluster is shown at the top of the subcluster. The red lines represent the gene expression level of the strobilus. The blue line shows the average values of the relative expression levels in each subcluster, and the gray lines represent the relative expression levels of each unigene. (**c**) The number of upregulated and downregulated unigenes for leaf vs. strobilus, leaf vs. root, leaf vs. stem, strobilus vs. root, strobilus vs. stem, and stem vs. root.

A total of 16,562 DEGs were found among the four organs of *P. chienii* (Figure 6a). The top 20 enriched pathways of 16,562 DEGs are shown in Figure S7. The 16,562 DEGs clustered into six subclusters through hierarchical cluster analysis (Figure 6b). The expression level of the strobilus organ was used as a control sample. The unigenes in subcluster 1 (3269 unigenes, accounting for 19.74%) had the highest expression level in the leaf, compared to those in the other three organs. The KEGG enrichment analysis of the unigenes in subcluster 1 found that most unigenes were involved in “photosynthesis-antenna proteins”, “photosynthesis”, and “carbon fixation in photosynthetic organisms”. In subcluster 2, 3762 unigenes (accounting for 22.71%) had the lowest expression level in the leaf, and most of these unigenes were enriched in “phenylpropanoid biosynthesis”, “flavonoid biosynthesis”, and “glutathione metabolism”. The unigenes in subcluster 3 (1549 unigenes, accounting for 9.35%) had the lowest expression level in the root, while in subcluster 5, 3460 unigenes (accounting for 20.89%) had a higher expression level in the root than in the other three organs. The unigenes involved in “flavonoid biosynthesis”, “circadian rhythm-plant”, and “brassinosteroid biosynthesis” were enriched in subcluster 3, and most of the unigenes in subcluster 5 were enriched in “diterpenoid biosynthesis”, “phenylpropanoid biosynthesis”, and “ribosome”. The unigenes in subcluster 4 (1614 unigenes, accounting for 9.75%) had a higher expression level in the strobilus than in the other three organs. The KEGG enrichment analysis of the unigenes in subcluster 4 revealed that most of them had functions in “cyanoamino acid metabolism”, “phenylpropanoid biosynthesis”, and “ribosome”. In subcluster 6, 2908 unigenes (accounting for 17.56%) had a higher expression level in the stem than in the other three organs. Unigenes in this subcluster functioned mostly in “diterpenoid biosynthesis”, “plant hormone signal transduction”, and “carotenoid biosynthesis”.

To identify the gene expression differences in the different organs (leaf vs. strobilus, leaf vs. root, leaf vs. stem, strobilus vs. root, strobilus vs. stem, and stem vs. root), we compared the paired combinations among the four organs of *P. chienii* to obtain the upregulated and downregulated unigenes in the latter relative to the former (Table S11). The details of the DEGs for leaf vs. strobilus, leaf vs. root, leaf vs. stem, strobilus vs. root, strobilus vs. stem, and stem vs. root are shown in Table S11. The largest differences were found for leaf vs. root, in which 9660 DEGs were determined, including 4092 upregulated unigenes and 5568 downregulated unigenes, and the number of annotated DEGs was also the greatest (Table S12, Figure 6c). These results indicate that a relatively large number of unigenes of the leaf and root of *P. chienii* might participate in the growth and development processes. To better explore the main biological functions and metabolic pathways, DEG enrichment analyses were performed in GO terms and KEGG pathways. In leaf vs. strobilus, leaf vs. root, leaf vs. stem, strobilus vs. root, strobilus vs. stem, and stem vs. root, the GO enrichment analysis indicated that 159, 243, 269, 135, 139, and 142 terms, respectively, were significantly enriched. The majority of categories were “catalytic activity” (GO: 0003824) and “metabolic process” (GO: 0008152) (Table S13). These two biological functions are important basic biological functions necessary for cellular life. In the enrichment of the KEGG pathways, 25, 30, 27, 22, 19, and 19 pathways were significantly enriched in the six paired combinations. Large numbers of DEGs were found in “phenylpropanoid biosynthesis” (ko00940) and “starch and sucrose metabolism” (ko00500) (Table S14). It is worth noting that “phenylpropanoid biosynthesis” (ko00940) was enriched in six paired combinations, which plays an important role in the chemical defense of conifer trees. “Phenylpropanoid biosynthesis” was further investigated. These enrichment analysis data provide insights into the metabolite biosynthesis in the four different organs of *P. chienii*.

### 2.8. Organ-Specific Expression Unigenes

We detected 28,901 organ-specific expression unigenes (31.07%) in the four organs of *P. chienii*. Of the four organs, the strobilus had the highest proportion of organ-specific unigenes (9124; 9.81%), followed by the stem (8140; 8.75%) and root (7244; 7.79%), whereas the leaf had the lowest proportion (4393; 4.72%) (Figure S6c). The number of strobilus-specific unigenes was greater than that of the other three organs, suggesting that the strobilus has unique characteristics as a reproductive organ. Our discovery of these organ-specific unigenes suggests that they are involved in physiological processes exclusive to certain organs. To explore the main biological functions of these organ-specific unigenes, GO enrichment analysis was performed on the four organs. In the strobilus, the most enriched GO terms were “transmembrane transport”, “single-organism transport”, and “single-organism localization”. The leaf organ had the least number of organ-specific unigenes, and these unigenes were mainly enriched in “single-organism metabolic process”, “oxidoreductase activity”, and “metabolic process”. The functions of these organ-specific unigenes are closely related to the biological characteristics and physiological statuses that are exclusive to certain organs. For instance, in the stem, the most enriched GO terms were “RNA polymerase II transcription factor activity, sequence-specific DNA binding”, “transmembrane transport”, and “transmembrane transporter activity”. Most of these highly enriched GO terms are closely related to the transport function of stem organ. Root-specific unigenes were mainly enriched in “oxidoreductase activity, acting on paired donors, with incorporation or reduction of molecular oxygen”, “iron ion binding”, and “heme binding”. Most of these highly enriched functions are closely related to the function of metal ion binding in root organ.

The KEGG pathway enrichment analysis of organ-specific expression unigenes revealed several significantly enriched pathways in the root, stem, leaf, and strobilus of *P. chienii*. In the strobilus, the most enriched KEGG pathways were “ribosome”, “cyanoamino acid metabolism”, and “arachidonic acid metabolism”. In the stem, the KEGG pathways of these unigenes were mainly enriched in “citrate cycle (TCA cycle)”, “pentose and glucuronate interconversions”, and “arginine biosynthesis”. As expected, the leaf-specific unigenes were mainly enriched in “carbon fixation in photosynthetic organisms”, “photosynthesis-antenna proteins”, “glyoxylate and dicarboxylate metabolism”, and “photosynthesis”. Root-specific unigenes were mainly enriched in “diterpenoid biosynthesis”, “ABC transporters”, and “phenylpropanoid biosynthesis”.

### 2.9. Discovery of Gene Families Related to Biotic/Abiotic Factors in P. chienii

Plants produce a number of TPSs for adaptation to various adverse environments [29]. Based on the annotation information, we identified 237, 67, 94, and 104 putative TPS unigenes for the root, stem, leaf, and strobilus, which included the TPS-d subfamily (79, 53, 40, and 42 putative unigenes for the root, stem, leaf, and strobilus), the TPS-c subfamily (one putative unigene for the strobilus and one for leaf), and unigenes with no subfamily identification (Tables S15 and S16). Those in the TPS-d subfamily were the most numerous and specific to gymnosperms. The majority of TPS sequences in *P. chienii* were homologues of *Taiwania cryptomerioides*, *Chamaecyparis obtusa*, and *Chamaecyparis formosensis*. In *P. abies*, several TPS genes have been demonstrated to increase insect and pathogen resistance [30]. The alignment of 25 TPS protein-coding sequences from the *P. chienii* transcriptome showed more than 70% sequence similarity to the TPSs from *P. abies* (GenBank accession numbers: AAO73863), and the highest sequence similarity was 84.38%.

**Figure 7:**
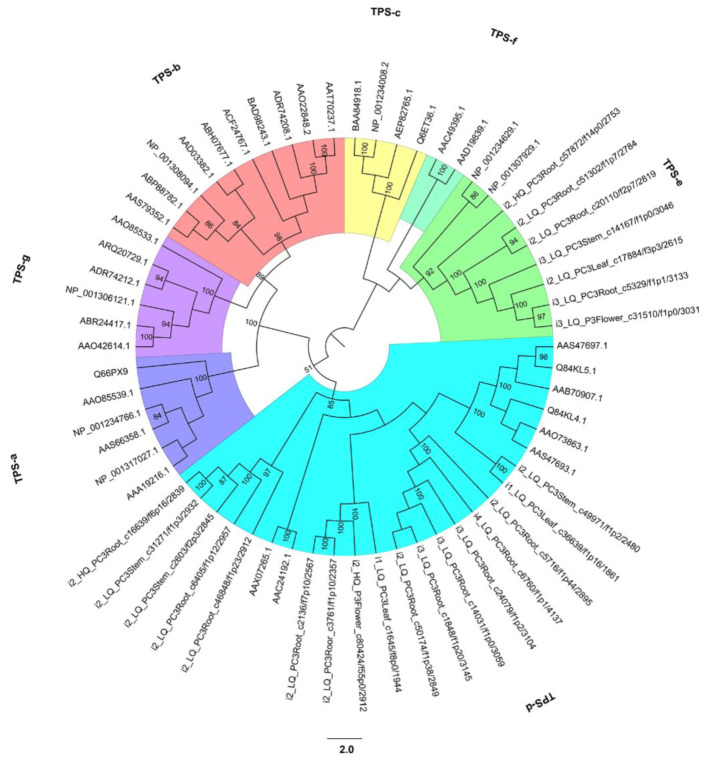
The phylogenetic analysis performed with 62 terpene synthase (TPS) sequences, including the filtered TPS protein-coding sequences of *P. chienii* (24 sequences) and other TPS sequences from gymnosperms and angiosperms (eight gymnosperm sequences and 30 angiosperm sequences).

We sought to further understand how these TPS sequences relate to a larger family comprised of both angiosperm and gymnosperm TPS sequences. Based on the filtered TPS protein-coding sequences of *P. chienii* and other TPS sequences from gymnosperms and angiosperms, the TPS phylogenetic tree showed that the sequences clearly fell into seven clades (from the TPS-a clade to the TPS-g clade). All TPS sequences from *P. chienii* were clustered in the TPS-d clade and TPS-e clade, which was consistent with the results of *Cupressus gigantea* [31]. The TPS-d clade possessed 17 TPS sequences from *P. chienii* and eight TPS sequences from other gymnosperms. The 17 TPS sequences of *P. chienii* contained in the TPS-d clade were also identified as belonging to the TPS-d subfamily based on the annotated results. Seven TPS sequences from *P. chienii* with no subfamily identification and two TPS sequences from *Solanum lycopersicum* were clustered in the TPS-e clade (Figure 7).

The CYP450 is one of the largest gene families in plant species and is important for the metabolism of xenobiotics [18]. CYP450 plays a crucial role in promoting plant development and protecting plants from various environmental stresses [32]. We identified 734, 362, 409, and 487 putative CYP450 unigenes in the root, stem, leaf, and strobilus, respectively (Table S17). The top 10 most abundant subfamilies of CYP450s included CYP725A1, CYP750A1, CYP82C4, CYP716B1, CYP87A3, CYP720B2, CYP716B2, CYP701A6, CYP734A1, and CYP76C2 (Figure S8). Among them, CYP725A1, CYP750A1, CYP716B1, CYP716B2, and CYP720B2 were gymnosperm-specific subfamilies. The most abundant CYP450 subfamily was CYP725A1, with a total of 196 sequences identified throughout the four organs of *P. chienii*. The CYP725A1 subfamily was the most abundant in root organ, with 146 sequences (Figure S8). The second most abundant subfamily was CYP750A1, with a total of 159 sequences identified throughout the four organs of *P. chienii*, and this subfamily was also the most abundant in root organ, with 66 sequences (Figure S8).

**Figure 8:**
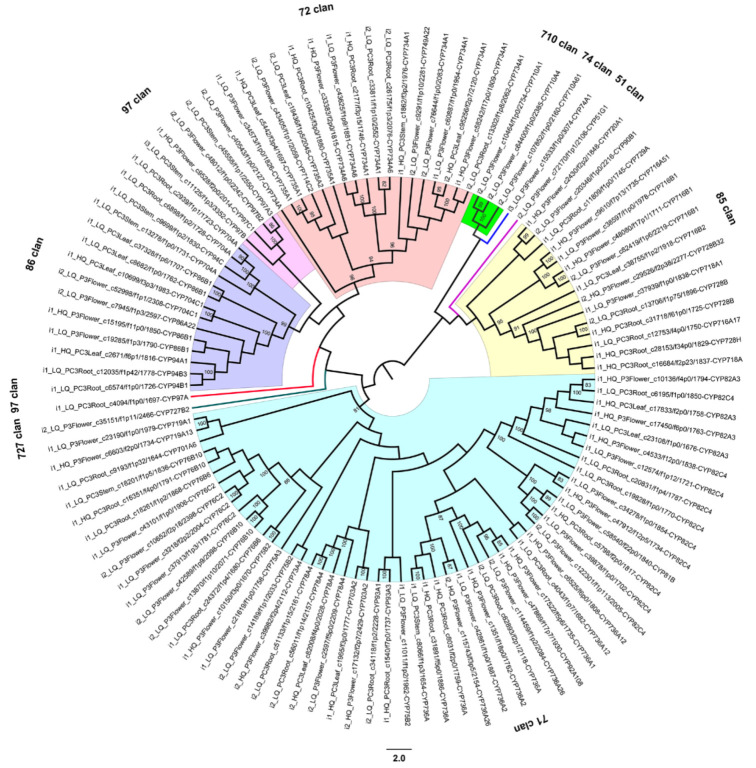
Phylogenetic analyses performed with 112 cytochrome P450 (CYP450) sequences of *P. chienii*. The classification of CYP450 protein-coding sequences was based on the reference sequences from a CYP450 database established by Nelson (https://drnelson.uthsc.edu/CytochromeP450.html) and the annotation results from the *P. chienii* transcriptome. Bootstrap support values over 80% are given.

The CYP450 phylogenetic tree with high bootstrap support >80% at most nodes showed that 112 putative CYP450 unigenes were classified into nine clans and 30 families and were divided into two categories: type A (71 clan) and non-type A (all other clans) (Figure 8). Within the NJ tree, 47.32% (53 unigenes) of the 112 putative CYP450 unigenes were type A and belonged to 11 families (CYP719, CYP701, CYP76, CYP75, CYP73, CYP78, CYP703, CYP93, CYP736, CYP92, and CYP82). The remaining 52.68% (59 unigenes) putative CYP450 unigenes were non-type A and were distributed among eight clans (727 clan, 97 clan, 86 clan, 72 clan, 710 clan, 74 clan, 51 clan, and 85 clan) and 18 families (CYP727, CYP97, CYP94, CYP86, CYP704, CYP734, CYP735, CYP714, CYP749, CYP710, CYP74, CYP51, CYP720, CYP90, CYP729, CYP716, CYP728, and CYP718), which included a more diverse group of unigenes. Type A CYP450s (71 clan) were identified to be related to the biosynthesis of secondary compounds. The non-type A CYP450s were involved in the metabolic pathways of primary products, plant hormones, and secondary products [33]. As a result, a large number of secondary metabolites were synthesized, and functioned as plant growth signals or protected plants from stresses [32].

The resistance and adaptation of plants to adverse environmental conditions are dependent upon the regulation of stress-related genes and gene families. HSPs, as ubiquitous molecular chaperones, are encoded by a multigene family, including five conserved families: small HSP (sHSP)/HSP20, HSP60, HSP70, HSP90, and HSP100 families [34]. In our study, a total of 223, 365, 165, and 464 putative HSP unigenes were identified in the root, stem, leaf, and strobilus, respectively. Among them, HSP70 is the most abundant HSP gene family, with a total of 851 unigenes identified throughout the four organs. The second most abundant family is HSP90, with a total of 248 unigenes identified throughout the four organs. The HSPs showed significant histological specificity in transcription. The identified putative HSP unigenes in leaf were generally less common than those in the other organs; in particular, HSP100 was not detected in leaf organ. The details of the HSP gene families are shown in Tables S15 and S18. In *Juglans regia*, *JrsHSP17.3* can enhance tolerance to abiotic stress, which enhances adaptability in plants [35]. In our study, among the 56 sHSP protein-coding sequences from the *P. chienii* transcriptome, 41 sequences showed a 45.97–85.94% sequence similarity to *JrsHSP17.3* (GenBank accession number: ALR81114.1). The expression patterns of the 56 sHSP unigenes showed that most of sHSP unigenes were expressed in at least one organ, while only six sHSP unigenes were not expressed in any organ. Thirty-one sHSP unigenes (55.36%) were predominantly expressed in the stem, and nine sHSP unigenes (16.07%) were highly expressed in the leaf, while the expression levels of the 24 sHSP unigenes were lower in the strobilus than in the other three organs (Figure S9).

### 2.10. Characterization of the Unigenes in Phenylpropanoid Biosynthesis Pathway

**Figure 9:**
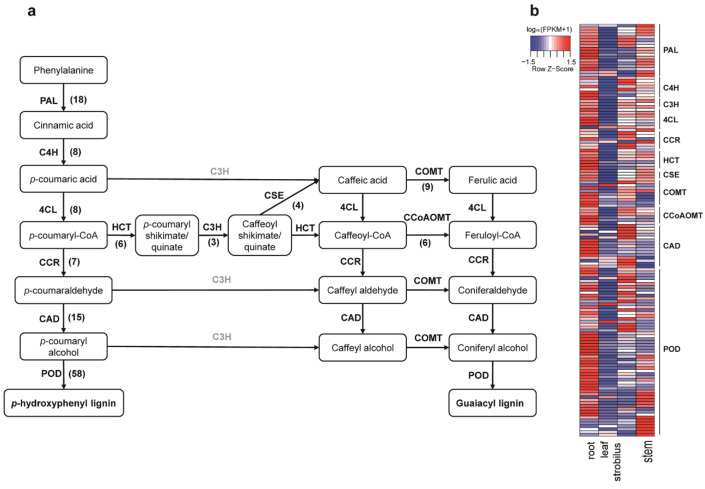
Biosynthesis pathway of phenylpropanoid in *P. chienii* and the differentially expressed genes (DEGs) involved in phenylpropanoid biosynthesis in four organs of *P. chienii*. (**a**) A simplified diagram of the phenylpropanoid biosynthetic pathway. Numbers in brackets represent DEG numbers; (**b**) a heatmap of the DEGs involved in phenylpropanoid biosynthesis in four organs. PAL: phenylalanine ammonia-lyase; C4H: *trans*-cinnamate 4-monooxygenase; 4CL: 4-coumarate CoA ligase; CCR: cinnamoyl-CoA reductase; HCT: shikimate *O*-hydroxycinnamoyltransferase; C3H: 5-*O*-(4-coumaroyl)-d-quinate 3′-monooxygenase; CAD: cinnamyl-alcohol dehydrogenase; POD: peroxidase; CSE: caffeoyl shikimate esterase; COMT: caffeic acid 3-*O*-methyltransferase; CCoAOMT: caffeoyl-CoA *O*-methyltransferase.

Based on the KEGG database, a total of 489 unigenes involved in the phenylpropanoid biosynthesis pathway were identified in four organs of *P. chienii* (Table S19, Figure 9a). For the phenylpropanoid biosynthetic pathway, 11 known enzymes were detected among the four organs, including PAL (phenylalanine ammonia-lyase, 100 unigenes), CYP73A/C4H (*trans*-cinnamate 4-monooxygenase, 16 unigenes), 4CL (4-coumarate CoA ligase, 51 unigenes), CCR (cinnamoyl-CoA reductase, 18 unigenes), HCT (shikimate *O*-hydroxycinnamoyltransferase, 27 unigenes), CYP98A/C3H (5-*O*-(4-coumaroyl)-d-quinate 3′-monooxygenase, 22 unigenes), CAD (cinnamyl-alcohol dehydrogenase, 58 unigenes), POD (peroxidase, 120 unigenes), CSE (caffeoyl shikimate esterase, 6 unigenes), COMT (caffeic acid 3-*O*-methyltransferase, 20 unigenes), and CCoAOMT (caffeoyl-CoA *O*-methyltransferase, 51 unigenes). Among them, 18 unigenes of PAL, eight of C4H, eight of 4CL, three of C3H, seven of CCR, six of HCT, six of CCOAOMT, four of CSE, nine of COMT, 58 of POD, and 15 of CAD were DEGs (Table S20). The expression patterns of these 142 DEGs associated with the phenylpropanoid biosynthesis pathway in four organs are shown in Figure 9b. Of these 142 DEGs, 82 DEGs were predominantly expressed in the root, including nine of PAL, five of C4H, two of C3H, six of 4CL, two of CCR, six of HCT, four of CSE, six of COMT, four of CCoAOMT, six of CAD, and 32 of POD. However, most of the DEGs (138 DEGs, accounting for 97.18%) had lower expression levels in the leaf than in the other three organs. In our study, we identified the complete pathway that forms the guaiacyl and *p*-hydroxyphenyl units, which are composed lignins of gymnosperms (Figure 9a).

### 2.11. Characterization of the Unigenes in Plant–Pathogen Interactions

**Figure 10:**
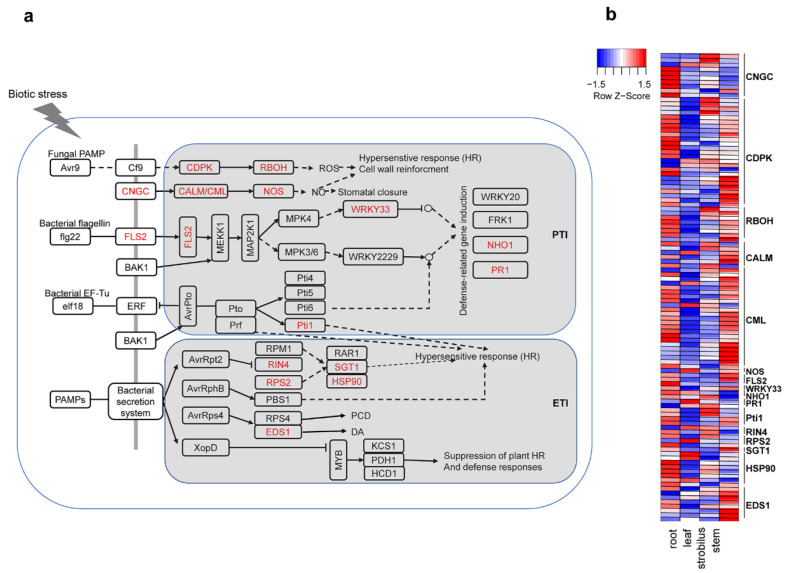
Plant–pathogen interactions in *P. chienii* and the differentially expressed genes (DEGs) involved in the plant–pathogen interactions in four organs of *P. chienii*. (**a**) A simplified diagram of the plant–pathogen interactions; (**b**) a heatmap of the DEGs involved in the plant–pathogen interactions in four organs. CNGCs: cyclic nucleotide-gated channel; FLS2: LRR receptor-like serine/threonine protein kinase FLS2; BAK1: brassinosteroid insensitive 1-associated receptor kinase; CDPK: calcium-dependent protein kinase; RBOH: respiratory burst oxidase; CALM: calmodulin; CML: calcium-binding protein CML; NOS: nitric-oxide synthase; PR1: pathogenesis-related protein; NHO1: glycerol kinase; RIN4: RPM1-interacting protein; RPS2: disease resistance protein RPS2; SGT1: suppressor of G2 allele of SKP1; EDS1: enhanced disease susceptibility 1 protein; HSP90: heat shock protein 90.

The conifer defense response under stress represents a significant adaptive mechanism [36]. Plants have evolved two main defense systems, pattern-triggered immunity (PTI) and effector-triggered immunity (ETI), which can be activated by plant pathogens [23]. In this study, some PTI- and ETI-related unigenes were detected (Figure 10a).

A total of 827 unigenes involved in the plant–pathogen interactions were detected in the four organs of *P. chienii* (Table S21). Several genes encoding PRRs were identified, including CNGCs (cyclic nucleotide-gated channel, 127 unigenes), FLS2 (LRR receptor-like serine/threonine protein kinase FLS2, 36 unigenes), and BAK1 (brassinosteroid insensitive 1-associated receptor kinase 1, 1 unigene). Among them, 10 unigenes of CNGCs and three of FLS2 were DEGs. In the PTI system of the plants, CDPK (calcium-dependent protein kinase, 184 unigenes), RBOH (respiratory burst oxidase, 94 unigenes), CALM (calmodulin, 39)/CML (calcium-binding protein CML, 66 unigenes), and NOS (nitric-oxide synthase, seven unigenes) were identified as important processes in the hypersensitive response (HR). FLS2 can activate the WRKY gene, which provides a strong response to pathogen infection and physical wounds in numerous plants [37,38]. In our study, we identified four WRKY33s, one of which was a DEG (Table S22). In addition, PR1 (pathogenesis-related protein 1, six unigenes) and NHO1 (glycerol kinase, 19 unigenes) are important transcriptional regulators of defense-related gene induction. In the ETI system of the plants, RIN4 (RPM1-interacting protein 4, three unigenes), RPS2 (disease resistance protein RPS2, 45 unigenes), SGT1 (suppressor of G2 allele of SKP1, nine unigenes), EDS1 (enhanced disease susceptibility 1 protein, 60 unigenes), and HSP90 (heat shock protein 90, 96 unigenes) were identified.

Among the 827 unigenes involved in the plant–pathogen interactions, 107 unigenes were DEGs. The expression patterns of these DEGs in the four organs are shown in Figure 10b. Of these 107 DEGs, 46 DEGs were predominantly expressed in the root, including six of CNGC, 12 of CDPK, five of ROBH, one of CALM, nine of CML, one of FLS2, one of NHO1, two of Pti1, one of RIN4, one of RPS2, five of HSP90, and two of EDS. Thirty-six DEGs were predominantly expressed in the stem, including one of CNGC, eight of CDPK, two of ROBH, two of CALM, 13 of CML, two of FLS2, one of WRKY33, one of SGT1, and six of EDS. Together, the expression levels of most DEGs in the plant–pathogen interactions were higher in the root and stem than that in the leaf and strobilus.

## 3. Discussion

### 3.1. PacBio Long Reads vs. Illumina Short Reads

With the development of sequencing technology, PacBio single-molecule real-time (SMRT) sequencing technology provides new insights into FL sequences. This technology is particularly suitable for non-model organisms without reference genome sequences [28]. In our study, using comprehensive analyses with PacBio Iso-Seq and Illumina RNA-Seq transcriptomic data, we provide an overview of the gene expression profiles in four organs of *P. chienii*, by taking advantage of the strong complementarity of these two data types.

A previous study of the *P. chienii* transcriptome used second-generation transcriptome sequencing technology (Illumina HiSeq2500 sequencing platform) [7]. The author obtained 78,192 unigenes, and the mean length and mean N50 were 622.78 and 1225 bp, respectively. In our study, we obtained 161,585 unigenes and obtained a longer mean length (1130 bp) and mean N50 (1588 bp) using Illumina RNA-Seq. Moreover, we selected four independent organ types, which maximized transcript diversity and provided an overview of the dynamic gene expression profiles in the different organs. The inclusion of more organs in library construction may be conducive to the discovery of rare and organ-specific unigenes. The number of unigenes from Illumina RNA-Seq was larger than the number of PacBio FL unigenes. However, the lengths of unigenes were mainly less than 2000 bp for the former, and longer than 2000 bp for the latter. These results indicate that PacBio Iso-Seq has a better ability to capture long sequences. However, a large number of small size unigenes less than 1000 bp may be missed using PacBio Iso-Seq, due to the technical limitations of size selection in the preparation of cDNA libraries for PacBio Iso-seq [39]. Thus, we used Illumina RNA-Seq data to effectively cover small size unigenes.

### 3.2. Functional Annotation

FL non-redundant sequence information improves the efficiency of functional gene prediction and annotation in plants. In this study, 85.38–94.48% of the PacBio Iso-Seq unigenes of four independent organ types were successfully annotated by at least one of seven public databases (Nt, Nr, Swiss-Prot, Pfam, KOG, GO, and KEGG) (Table 1). These percentages of annotated unigenes based on PacBio Iso-Seq data were higher than for the Illumina RNA-Seq analysis (79.2–81.79%), indicating that PacBio Iso-Seq data could provide more accurate and effective information for *P. chienii* transcriptomes. A few unigenes obtained in this study were not annotated in any of the seven public databases. There are several possible reasons: first, there may be a lack of genomic reference information on Taxaceae; second, the unannotated unigenes likely contain non-coding RNA or belong to untranslated regions; third, the unigenes without annotations may be short sequences of absent protein domains. Unannotated unigenes may also be considered novel or species-specific transcript sequences. Our data enriched transcript resources of *P. chienii* offered good performance for discovering novel or uncharacterized transcripts and genes. The GO annotation analyses showed that “metabolic process”, “cellular process”, “binding”, “catalytic activity”, “cell”, and “cell part” comprise the majority of subcategories, indicating that the organs of *P. chienii* experience active cell metabolism to accumulate sufficient nutrition for the future growth and development of the organs. These GO terms are consistent with the previously investigated *Taxus cuspidata* transcriptome [25] and the *Taxus mairei* transcriptome [40]. The KEGG annotation analysis showed that “carbohydrate metabolism” and “signal transduction” are the top two pathways with the most abundant unigenes. “Carbohydrate metabolism” can prevent plant cell damage or cell death under low temperatures [41], and “signal transduction” plays an important role in stress response [42].

### 3.3. TFs, LncRNAs, and AS Analysis

FL non-redundant sequences provide much genetic information for transcriptional regulation and posttranscriptional regulation, such as TFs, lncRNAs, and AS events. TFs regulate gene expression via interactions with themselves and other proteins under plant stress response [43]. In our study, we identified 1678, 2432, 1706, and 2497 TFs for the root, stem, leaf, and strobilus, respectively, belonging to 66 different families, which were comparable to those of another conifer species *T. cuspidata* (1940 TFs), and the model organism *A. thaliana* (2357 TFs) [25]. C2H2, C3H, bHLH, and bZIP were the most represented TF families in all four organs of *P. chienii*. The phenomenon of the C2H2 family being the most abundant might be due to a greater number of C2H2 gene duplication events occurring in the *P. chienii* genome during the evolutionary process, which might have special functions for these genes. C3H is member of a large family of TFs that modulate the expression of downstream stress-responsive genes in plants [44]. bZIP TFs regulate the processes of stress signaling, flower development, and seed maturation [45]. bHLH TFs are one of the largest regulatory protein families, and are widely distributed in eukaryotes, participating in multiple physiological processes [46]. The expression levels of most DEGs of C2H2, C3H, bHLH, and bZIP families in the root and stem were higher than those in the leaf and strobilus, indicating that these TFs may play a crucial role in the survival of the root and stem. However, MYB-related, NAC, GARP-G2-like, and MYB were more highly expressed in the strobilus than in the other three organs. The expression analysis shows that the TF families exhibit functional differentiation.

LncRNAs, a class of nonprotein-coding unigenes longer than 200 nt, are important components of the gene expression regulators functioning in biological processes and plant stress responses [47,48]. The characteristics and genetic patterns of lncRNAs in *P. chienii* are still unclear. Unlike coding RNAs, lncRNAs lack homology among closely related species. As a result, the information regarding predicted non-coding RNAs for one species is not necessarily useful for other species, which makes prediction and annotation challenging [49]. Recently, increasing numbers of studies have focused on characterizing the functions of lncRNAs in plant species, thereby providing a basis for understanding the functions of lncRNAs in response to environmental change [50]. The more organ types that are used, the more lncRNAs that can be discovered in this species [51]. In our study, lncRNAs were identified in four organ types, with the most in stem and the fewest in leaf. The mean length of lncRNAs (1382 bp) was about double the length of that identified in red clover (*Trifolium pratense* L.) (665.39 bp) [52]. Additionally, consistent with previous research, the lncRNAs were shorter than the protein-coding mRNAs [50]. Information on these lncRNAs could represent a useful resource for further research on the potential functions and regulatory mechanisms of lncRNAs in *P. chienii*.

AS is a crucial regulatory mechanism, by which multiple proteins can be produced from a single gene, making it a key factor in greatly increasing the diversity of the transcriptome and proteome of many eukaryotes [53,54]. AS represents a posttranscriptional regulatory mechanism that can affect gene expression [55]. During different developmental programs, or under various environmental stresses, the occurrence of AS in plants is high, which has led to an increasing number of studies focusing on revealing the regulatory mechanisms of AS by deep sequencing [56]. Using transcriptome sequencing technologies, AS analysis has been performed in *Arabidopsis* [56], maize (*Zea mays*) [57], and soybean (*Glycine max*) [58]. PacBio long reads contain all of the information originating from a single RNA molecule, giving the data the ability to detect the complexity of an AS event of a species without a reference genome [32]. In our study, we identified 461, 475, 559, and 430 AS events in the root, stem leaf, and strobilus, including RI, A3, A5, SE, AF, AL, and MX AS event types. RI events were found to account for the largest proportion of AS events, which is considered reasonable in terms of patterns of AS in plants [56]. However, the number of RIs was estimated to be 34.18% for all AS events in *P. chienii*. This proportion is less than that in other published studies on plants (~40%) [56]. We suspect that our study underestimates the actual percentage of AS events, since we only used four organ types, corresponding to the same developmental stage under natural growth conditions. We expect that more AS events will be revealed when more organ types are subject to transcriptome analyses during more developmental stages under environmental stress conditions.

### 3.4. DEGs and Organ-Specific Unigenes

A total of 16,562 DEGs were identified among the four organs. It is worth noting that the DEGs and expression profiles we detected come from different organs, not different tissues. KEGG enriched analysis was performed and the top three enriched KEGG pathways were “phenylpropanoid biosynthesis”, “flavonoid biosynthesis”, and “diterpenoid biosynthesis”. The emergence of the phenylpropanoid pathway is one of the important adaptive mechanisms. Phenylpropanoids are precursors to a wide range of compounds, such as isoflavonoids, flavonoids, and stilbenes. These compounds play important roles in plant defense against biotic and abiotic stresses [59]. Flavonoids, a group of powerful antioxidant compounds, were synthesized, starting with the phenylpropanoid pathway. Nakabayashi et al. reported that flavonoids, including flavonols and anthocyanins, have a strong radical scavenging activity, and can protect plants from oxidative and drought stress [60]. During jujube tree (*Ziziphus jujuba*) infection, the genes involved in phenylpropanoid biosynthesis and flavonoid biosynthesis were significantly upregulated [61]. Diterpenoid compounds are important conifer defense chemicals against herbivores and pathogens [62]. Our results demonstrate that these DEGs play a vital role in enabling adaptation to diverse environments and regulating the growth and development of four different organs in *P. chienii*.

Organ-specific unigenes were enriched in specific molecular functions and metabolic pathways in different organs, which is consistent with the results published for other plants [63,64]. Photosynthesis-related pathways, such as “carbon fixation in photosynthetic organisms”, “photosynthesis-antenna proteins”, and “photosynthesis”, were enriched in leaf organ, which are related to the biological characteristics of leaf organ. Stem-specific unigenes were mainly enriched in the “transmembrane transport” GO term and the “pentose and glucuronate interconversions” pathway. Some studies have suggested that “transmembrane transport” may participate in drought and salt stress responses [42,65], and “pentose and glucuronate interconversions” is involved in the response to anthracnose infection in tea plant (*Camellia sinensis*) [66]. Root-specific unigenes were significantly enriched in the “ABC transporters” pathway. ABC transporters are involved in a variety of biological processes, enabling plants to adapt to diverse environmental conditions and respond to biotic and abiotic stresses [67]. The absence of ABC transporters affects the expression of many genes related to suberin formation and cuticle metabolism in roots [68]. Strobilus-specific unigenes were significantly enriched in the “arachidonic acid metabolism” and “cyanoamino acid metabolism” pathways. Arachidonic acid is a fungal elicitor that induces a hypersensitive response [69]. “Cyanoamino acid metabolism” might be implicated in H_2_S-dependent drought tolerance in wheat (*Triticum aestivum* L.) [70]. These significantly enriched molecular functions and pathways of organ-specific unigenes indicate the specific development and environmental response patterns of their corresponding organs. A hierarchical cluster analysis showed the organ-biased expression patterns. The unigenes in different subclusters were involved in the development or the environmental response of the corresponding organs. Together, the expression profiles for genes involved in responses to the environment and growth maintenance were shown to be distinct and organ-specific, and would have significant implications regarding the organ development of *P. chienii*.

### 3.5. Gene Families Related to Biotic/Abiotic Factors in P. chienii

Terpenoids play a significant role in the chemical and physical defenses in coniferous trees and are produced by the TPS gene family [62]. In our study, we identified 237, 67, 94, and 104 putative TPS unigenes in the root, stem, leaf, and strobilus of the *P. chienii* transcriptome, most of which share high sequence similarity with the TPS sequences from other plants. The identified unigenes of TPS were the most numerous in root organ, suggesting that the TPS gene family may be involved in the defense against root-attacking soil-borne organisms. These numbers are comparable with the numbers of TPSs in angiosperm genomes and other coniferous species (40 in *A. thaliana* [71], 47 in poplar (*Populus trichocarpa*) [72], 113 in *Eucalyptus* [73], 69 in grapevine (*Vitis vinifera*) [74], 83 in white spruce (*P. glauca*) [75], 43 in *Platycladus orientalis*, and 93 in loblolly pine (*Pinus taeda*) [76]).

Based on the annotation results and phylogenetic analysis, the *P. chienii* TPS sequences belong to the TPS-c, TPS-d, and TPS-e subfamilies, which is consistent with the previously constructed phylogeny of the gymnosperm TPS family [77]. The majority of gymnosperm TPSs belong to the gymnosperm-specific TPS-d subfamily, and fewer gymnosperm TPSs fall into the subfamilies TPS-c and TPS-e [77]. TPS-c contains copalyl diphosphate synthases (CPS), bifunctional copalyl diphosphate synthases/kaurene synthases (CPS/KS), and other diterpene synthases. TPS-e contains kaurene synthases (KS). The gymnosperm-specific TPS-d subfamily has high functional plasticity, and its great diversity of produced terpenoid compounds may contribute to its defense against stress [78]. The synthesis of the majority of terpene compounds has evolved in plants as a mechanism improved the adaptability of each species to their local ecological niche [77]. The functional characterization of coniferous TPS gene family has been a research hotspot [30,78]. For instance, the expression of many TPS genes involved in volatile emissions and oleoresin defenses can be induced by pathogen infection or herbivore attacks in conifers [79], suggesting that TPS genes have important functions in the defense systems of conifers.

The CYP450 superfamily is one of the largest gene families in plants and mediates reactions involving both primary and secondary metabolites [80]. CYP450 families are involved in various biosynthesis pathways, such as the flavonoid pathway, terpene pathway, salicylic acid pathway, and phenylpropanoid pathway. However, it is difficult to determine the metabolic functions of CYP450s because these enzymes occur in very low quantities and are labile in plants [81]. Therefore, we used transcriptome sequencing to classify CYP450s into unique families and accurately predict their functions. In this study, we identified 734, 362, 409, and 487 putative CYP450 unigenes in the root, stem, leaf, and strobilus respectively. Among the four organs, the unigenes of CYP450 in root were the most abundant. Some of the known gymnosperm-specific CYP450 subfamilies, CYP725A, CYP750A, CYP716B, and CYP720B, were discovered in *P. chienii*. CYP725A1 is the largest non-type A subfamily, and we identified 146, 16, eight, and 26 putative CYP725A1 unigenes in the root, stem, leaf, and strobilus, respectively. CYP725A1 is a stress-responsive gene, and the molecular regulation of stress-responsive gene expression plays a significant role in the plant response to various stresses [82]. CYP750A1 was found to be the largest type A subfamily, which is consistent with the evidence for *T. chinensis* [33]. We identified 66, 28, 27, and 38 putative CYP750A1 unigenes in the root, stem, leaf, and strobilus, respectively. For the root, stem, leaf, and strobilus, we identified 16, 14, 14, and 19 putative CYP716B1 unigenes and 15, five, 15, and 12 putative CYP716B2 unigenes in *P. chienii*. The only confirmed function of the CYP716B gene is the taxoid 9α-hydroxylase in *Ginkgo biloba* [83]. We identified 30, seven, seven, and 11 putative CYP720B2 unigenes in the root, stem, leaf, and strobilus, respectively. In conifers, CYP720B is involved in the formation of diterpene resin acids, which is a major part of the oleoresin defense in conifers [84]. We also identified the CYP73A and CYP98A subfamilies, which are involved in phenylpropanoid biosynthesis and catalyze the first two hydroxylation reactions in the phenolic rings of phenylpropane units. These numbers of CYP450 unigenes in the *P. chienii* transcriptome are of the same order of magnitude as those in other plant genomes, such as 307 in white spruce (*P. glauca*) [75], 272 in *A. thaliana*, 455 in rice (*Oryza sativa*), and 312 in poplar (*P. trichocarpa*) [85].

The CYP450 phylogenetic tree analysis provided information about the functional evolution and hinted at some deeper relationships of the CYP450 gene family in *P. chienii*. For example, 86 clan was adjacent to 97 clan. 85 clan, 51 clan, 74 clan, and 710 clan clustered into a single lineage. These results are consistent with the NJ tree of 142 plant CYP450s constructed by Nelson and Werck-Reichhart [80]. Within the CYP450 phylogenetic tree, 112 putative CYP450 unigenes were classified into nine clans, including four multi-family clans (71 clan, 72 clan, 85 clan, and 86 clan) and five single-family clans (51 clan, 74 clan, 97 clan, 710 clan, and 727 clan). The single-family clans restrict themselves from gene duplication, and the enzymes they include are highly conserved with essential functions. These clans are most likely to undergo negative selection [86]. CYP51 is considered to be one of the most conserved and the oldest eukaryotic CYP450s [80]. CYP74 is an atypical CYP450 that is engaged in the formation compounds with signaling or antimicrobial functions [87]. The diversification of CYP450s in multi-family clans parallels the adaptive evolution of land plants. Particularly, the CYP71 clan has expanded rapidly, representing by itself about 50% of plant CYP450s. Therefore, the CYP71 clan has a great diversity of functions, including being involved in the biosynthesis of lignin, cutin, and sporopollenin [80].

HSPs were originally well-documented in plant responses to high-temperature conditions. However, increasing numbers of investigations have confirmed that HSPs are induced by various abiotic stresses and are involved in plant growth and development [88]. In our study, 56, 23, 851, 248, and 39 unigenes were identified as members of sHSP, HSP60, HSP70, HSP90, and HSP100 families, respectively. HSP70 is the most abundant HSP gene family and is a housekeeping gene functioning in protein folding and protein quality control, followed by HSP90, with a key role in protein degradation and trafficking, cell cycle control, and signal transduction networks [21,89]. The features and functions of HSPs have been widely studied in many plants [90]. For example, almost all the *CsHSP* genes of *C. sinensis* are expressed in one or more organs, and are strongly induced under drought and heat stress [91]. The expression of loblolly pine (*P. taeda*) *HSP* genes shows distinctive responses associated with acclimation [92]. In *Arabidopsis*, the expression of *HSP90* is significantly induced under salinity, cold, heat, and heavy metal stress [93,94]. The expression of *Chenopodium quinoa Cqhsp70s* enhances drought tolerance [95]. Similarly, the functions of sHSP have been widely confirmed. For instance, *Salix suchowensis Ssu-sHsps* may participate in plant development and stress tolerance by interacting with TFs and functional genes [96]. The expression patterns of the 56 sHSP unigenes of *P. chienii* indicate that 31 sHSP unigenes (55.36%) are predominantly expressed in the stem, indicating that they may have potential roles in stem development and stress response.

### 3.6. Characterization of the Unigenes in Phenylpropanoid Biosynthesis Pathway

*P. chienii* is highly adaptable to the diverse environments involved in biotic and abiotic factors [5,6]. Metabolites originating from the phenylpropanoid pathway play an important role in conifer defense [97]. We characterized the key genes in the synthesis of the defensive compounds from the phenylpropanoid biosynthesis pathway. These findings are of great significance to the study of the adaptive mechanisms in *P. chienii*.

Several genes involved in the phenylpropanoid pathway produce physical and chemical barriers against stress, including the formation of lignin and other phenylpropanoids. In the transcriptome sequences of *P. chienii*, we found FL transcriptome sequences for all key genes in the phenylpropanoid pathway, except for ferulic acid 5-hydroxylase (F5H/CYP84). F5H is necessary to produce syringyl lignin. However, conifers do not produce syringyl lignin [98]. Therefore, F5H is not expected to be present in *P. chienii*. In the phenylpropanoid pathway, we identified other CYP450s, including C3H/CYP98A and C4H/CYP73A. The CYP450 enzyme is the most common derivative involved in the detoxification mechanism of phenylpropanoid biosynthesis. Additionally, of the 142 DEGs, 82 DEGs were predominantly expressed in the root. Therefore, the root constitutes the main organ of phenylpropanoid synthesis and accumulation. Most of the DEGs in phenylpropanoid biosynthesis had relatively low expression levels in the leaf compared to the other three organs, which is consistent with the results for white spruce [99].

The enzymes in the phenylpropanoid pathway of conifers and their involvement in defense mechanisms have been characterized. POD is related to the polymerization of lignin and the oxidation of phenolic compounds. In *P. abies*, POD conferred resistance against infection by the pathogen *Pythium dimorphum* [100]. In Sitka spruce (*P. sitchensis*), several known enzymes (including PAL, C3H, C4H, 4CL, CCR, COMT, CCoAOMT, and CAD) involved in phenylpropanoid biosynthesis participate in plant defense against insects or wounding [101]. In *Larix olgensis* Henry, seven DEGs (including 4CL, CCoAOMT, CCR, CAD, PAL, C4H, and POD) related to lignin synthesis were demonstrated to increase disease resistance via cell wall thickening [102]. Lignins from gymnosperms are composed mostly of guaiacyl units, and minor amounts of *p*-hydroxyphenyl units [103]. In our study, we identified the complete pathway that forms guaiacyl and *p*-hydroxyphenyl units (Figure 9). Lignin is essential for the structural integrity of cell walls and the strength and stiffness of the root and stem. Lignin waterproofs the cell walls, enabling water solutes to be transported through the vascular system [97].

### 3.7. Characterization of the Unigenes in Plant–Pathogen Interactions

Conifers are often attacked by pathogens and have developed sophisticated resistance mechanisms to protect themselves. In this study, some PTI- and ETI-related unigenes were detected (Figure 10), indicating that the PTI and ETI defense systems were employed in *P. chienii*.

Pathogenic signals are transmitted to the cytoplasm by identifying CNGCs, FLS2, and BAK1. The PTI response is then initiated and amplified. In our study, PTI-related unigenes were identified, including CDPK, RBOH, CALM/CML, NOS, FLS2, WRKY33, NHO1, PR1, and Pti1. CALMs are crucial for responses to several biotic and abiotic stresses in plants [104,105]. The expression level of tobacco NtCaM13 was increased in TMV-infected leaves [104]. In this study, 39 unigenes encoding CALMs were identified. Within the cell nucleus, expression of the defense-related gene WRKY33 and its downstream pathogen-resistance genes NHO1 and PR1 were detected. In general, PTI is enough to defend against most pathogens. However, some pathogens have evolved effector proteins to suppress PTI, which can be overcome by triggering ETI [106]. PTI and ETI share signaling components and outputs, but ETI amplitudes are higher and more persistent, often leading to HR [107]. In our study, several ETI-related unigenes were identified, including RIN4, RPS2, HSP90, SGT1, and EDS1. RPS2 was able to induce disease resistance, and EDS1 could accelerate programmed cell death [108]. The expression levels of most DEGs in the plant–pathogen interactions are higher in the root and stem than that in the leaf and strobilus, suggesting that root and stem organs have a stronger ability to respond to stress.

## 4. Materials and Methods

### 4.1. Plant Materials and RNA Isolation

The root, stem, leaf, and strobilus were collected from *P. chienii* in 2018 at Bijia Mountain (114°09′42″ E, 26°30′37″ N, 1290 m a.s.l.), Jiangxi Province, China. Four samples of different organs were collected from the same plant. Fresh and healthy samples were washed with purified water and dried with paper towels. All samples were cut into pieces and stored immediately in RNAfixer (BioTeke, Shanghai, China). After collection, all samples were stored at −20 °C in a refrigerator until further use.

Total RNA extraction of each sample was performed using a RNAprep Pure Plant Kit, following the protocol of the manufacturer (TianGen, Beijing, China). The purity and concentration of the extracted RNA were determined using a NanoDrop spectrophotometer (Thermo Scientific, Wilmington, DE, USA) and Qubit 2.0 Fluorometer (Invitrogen, Life Technologies, Carlsbad, CA, USA), respectively. RNA integrity was assessed using an Agilent 2100 Bioanalyzer (Agilent Technologies, Santa Clara, CA, USA). High quality RNA with RNA integrity number (RIN) > 8.0 was used for cDNA synthesis and library construction. In total, 1 μg RNA of each sample was used for cDNA library construction.

### 4.2. PacBio Library Construction and Sequencing

Total RNA for each of the four organs was constructed library separately according to the PacBio Isoform Sequencing (Iso-Seq) experimental protocol. Poly(A) mRNA was isolated from total RNA using Dynabeads Oligo (dT) magnetic beads (Dynal, Life Technologies, Carlsbad, CA, USA). First-strand cDNA was synthesized from poly(A) mRNA using a Clontech SMARTer PCR cDNA Synthesis Kit (Clontech, Mountain View, CA, USA). Then, after optimization of the PCR cycles, large-scale PCR amplification was used to synthesize second-strand cDNA. Size selection was performed with a BluePippin Size Selection System (Sage Science, Beverly, MA, USA) for each sample. Unfiltered fragments and >4 kb fragments were equally mixed and used to construct the SMRTbell library with the SMRTBell Template Prep Kit (Pacific Biosciences, Menlo Park, CA, USA). SMRTbell libraries were sequenced on the PacBio Sequel platform (Pacific Biosciences, Menlo Park, CA, USA) using V2 chemistry with 10 h movies. A total of eight SMRT cells were used for sequencing.

The PacBio Iso-Seq FL transcriptome data were deposited in the Sequence Read Archive (SRA) of NCBI as follows: root: SRR11715805; stem: SRR11715804; leaf: SRR11715803; strobilus: SRR11715802.

### 4.3. Illumina Library Construction, Sequencing, and De Novo Assembly

The Illumina library for each organ was constructed using a NEBNext Ultra™ RNA Library Prep Kit (NEB, Ipswich, MA, USA), according to the manufacturer’s protocol. Sequencing was performed on the Illumina NovaSeq platform, generating paired-end (PE) reads with lengths of 150 bp. Illumina RNA-Seq raw reads were processed using in-house Perl scripts. Clean reads were obtained by removing the adapter reads, reads with more than 10% ambiguous bases ‘N’, and low-quality reads (Qphred ≤ 20 base with more than 50%) from the raw reads. Clean reads were de novo assembled with Trinity v.2.4.0 [109] using the following parameters: min_kmer_cov: 2, as well as the remaining default parameters.

The Illumina RNA-Seq transcriptome raw data were deposited in the SRA of NCBI as follows: root: SRR11715801; stem: SRR11715800; leaf: SRR11715799; strobilus: SRR11715798.

### 4.4. Preprocessing of the PacBio Iso-Seq Data

PacBio Iso-Seq raw data were processed with the SMRTlink 5.1 software (http://www.pacb.com/products-and-services/analytical-sofware/smrt-analysis/). Subreads were obtained from the raw reads using the following parameters: min_length 200, min_readscore 0.65. CCSs were obtained from the subreads by self-correction using the following parameters: min_length 50, max_drop_fraction 0.8, no_polish TRUE, min_zscore −9999.0, min_passes 2, min_predicted_accuracy 0.8, max_length 15,000. The CCSs were then classified into FLNC and non-full-length (NFL) reads according to whether or not the 5′-primer, 3′-primer, and poly(A) tail were observed. FLNC reads were performed no isoform-level clustering with the ICE algorithm [110] to obtain FL consensus sequences. The FL consensus sequences were polished by NFL reads using the Arrow algorithm (parameters: hq_quiver_min_accuracy 0.99, bin_by_primer false, bin_size_kb 1, qv_trim_5p 100, qv_trim_3p 30) to obtain HQ, FL, and polished consensus isoforms. Because PacBio reads have a higher frequency of nucleotide errors than Illumina short reads, the polished consensus isoforms were corrected by Illumina RNA-Seq data using the LoRDEC tool [111]. Finally, redundant sequences were removed with CD-HIT (parameters: -c 0.95 -T 6 -G 0 -aL 0.00 -aS 0.99) to obtain the final transcripts (unigenes) [112]. The final transcripts of multiple organs were merged, and the redundant transcripts were removed based on CD-HIT (parameters: -c 0.95) to obtain the reference transcriptome sequences for *P. chienii*. The completeness of the transcriptome sequences was evaluated using the BUSCO [113] method with the Embryophyta (ODB9) core gene dataset.

### 4.5. Functional Annotation

The final obtained transcript sequences (unigenes) of the four organs were mapped to seven databases to obtain function annotation information. The NCBI Nr (https://www.ncbi.nlm.nih.gov/protein/), KOG (ftp://ftp.ncbi.nih.gov/pub/COG/KOG/), KEGG (http://www.genome.jp/kegg/), and Swiss-Prot (https://www.uniprot.org/uniprot/) databases annotations were performed using DIAMOND v0.8.36 [114] with an *E*-value threshold of 1.0 × 10^−5^. NCBI Nt database annotation was performed using ncbi-blast-2.7.1+ with an *E*-value threshold of 1.0 × 10^−5^. Pfam (https://pfam.xfam.org) database annotation was performed using Hmmscan of the HMMER 3.1 package (http://hmmer.org/) [115]. GO (http://www.geneontology.org/) annotations were performed based on the protein annotation information from the Pfam database, using Blast2GO (http://www.blast2go.com) [116] and a script. Based on the KEGG database annotation information, the unigenes involved in the phenylpropanoid biosynthesis pathway and the plant–pathogen interactions were identified in the four organs of *P. chienii*.

### 4.6. Prediction of CDSs, TFs, and LncRNAs

The ANGEL pipeline was used to predict the CDSs from cDNAs [117]. Firstly, the confident protein sequences of this species and closely related species were used for ANGEL training. Then, the given sequences were predicted using ANGEL. iTAK software was used to identify the plant TFs [118].

Unigenes with lengths greater than 200 nt were selected as lncRNA candidates and further screened via four computational approaches. To obtain a set of high confidence lncRNAs, we calculated the coding potential of each unigene using a series of filtering steps: firstly, PLEK (a predictor of lncRNAs and messenger RNAs based on an improved k-mer scheme) [119] and Coding-Non-Coding Index (CNCI) [120] were employed to categorize the protein-coding and non-coding unigenes and assess coding potential; secondly, the Coding Potential Calculator (CPC) [121] was used to search the sequences via the NCBI eukaryote protein database (*E*-value = 1.0 × 10^−10^); finally, we translated each unigene in all three possible frames and identified the known protein family domains documented in the Pfam database according to a Hmmscan homology search with the default parameters of -E 0.001 -domE 0.001. Any unigene with known protein family domains was excluded. As a result, the intersections of predictions of the four filtering tools were considered as the potential lncRNAs of *P. chienii*.

### 4.7. AS Analysis

We used PacBio Iso-Seq data to reconstruct FL UniTransModels for each of the four organs of *P. chienii*. Error-corrected non-redundant transcripts were processed with the Coding GENome reconstruction Tool (Cogent v3.1, https://github.com/Magdoll/Cogent) following the parameters: --dun_use_partial. Firstly, the k-mer profiles of transcripts were created using Cogent by calculating the pairwise distances. Secondly, these transcripts were clustered into families based on their k-mer similarities. Thirdly, the transcript families were further reconstructed into UniTransModels with the de Bruijn graph method. Finally, transcripts were aligned to the UniTransModels using the Genome Mapping and Alignment Program (GMAP-2017-06-20) [122]. AS events were identified using SUPPA (https://github.com/comprna/SUPPA) [123] with default parameters. Different AS event types were identified from the alignments and classified into RI, A3, A5, MX, AL, alternative first exons (AF), and skipping exon (SE).

### 4.8. Gene Expression Quantification, DEGs, and Functional Enrichment Analysis

The clean Illumina reads of each organ sample were mapped to the reference transcriptome sequences using Bowtie 2 [124] of the RSEM (RNA-seq by expectation maximization) software [125] with the end-to-end, sensitive mode and other default parameters, and the readcount value of each unigene for each organ was obtained. Then, all readcounts were normalized to the FPKM [126]. The expression level of each unigene in each sample was determined by estimating the FPKM using RSEM.

The readcount for each unigene in each organ was adjusted with the EdgeR program [127] by one scaling normalized factor. The DEG analysis between each pair combination of different organs (leaf vs. strobilus, leaf vs. root, leaf vs. stem, strobilus vs. root, strobilus vs. stem, and stem vs. root) was performed using the DEGseq of the R package [128] with Poisson’s distribution. The *p* value was adjusted using the *q* value [129] with the Benjamini–Hochberg approach for controlling the false discovery rate (FDR). Unigenes with *q* value < 0.005 and |log_2_(fold change)| > 1 were considered to be the DEGs. The fold change represents the ratio of the expression level between each two organ samples. Heatmaps were plotted using the gplots 3.0.3 package in R software. Based on the DEGs, a hierarchical cluster analysis was performed using the cluster 2.0.5 package in R software. Unigenes expressed only in one organ and not in any other organ were identified as organ-specific unigenes.

GO and KEGG enrichment analyses were performed to identify significantly enriched biological functions and metabolic pathways in DEGs compared against the whole transcriptome background. The GO enrichment analysis of DEGs was implemented via the GOseq R package based on Wallenius non-central hyper geometric distribution [130], in which gene length bias was corrected. GO terms with adjusted *p* values (*q* values) < 0.05 were considered significantly enriched. The KEGG pathway enrichment analysis of the DEGs was performed using KOBAS (2.0) [131].

### 4.9. Identification of the Gene Families Related to Biotic/Abiotic Factors

Based on functional annotations from publicly available databases (Nr, Swiss-Prot, Pfam, and KOG), the biotic/abiotic factor-related gene families were identified in *P. chienii*. The number of unigenes annotated for known biotic factor-related gene families (TPS and CYP450) and abiotic factor-related gene families (HSP) were compared among the four organs of *P. chienii*. The majority of biotic factor-related genes were of the CYP450 gene family. Therefore, the CYP450 and HSP gene families were further investigated. The top 10 most abundant subfamilies of CYP450 in all organs were identified. Some sequences did not feature subfamily identification and were thus not counted. Such genes identified in this study were aligned with the genes conferring resistance in previous studies to assess their homology and similarity in *P. chienii* with an *E*-value threshold of 1.0 × 10^−10^.

### 4.10. Phylogenetic Analyses

TPS and CYP450 phylogenetic trees were constructed using the neighbor-joining (NJ) method with the Poisson correction model and 1000 bootstrap replications in MEGA 5.10 [132]. First, the TPS protein-coding sequences were obtained from the *P. chienii* transcriptome. Second, redundant sequences (95% similarity) were removed, and only the longest protein-coding sequences were retained. Third, sequences with less than 500 aa and incomplete domains were removed. Fourth, 38 TPS protein sequences of other plants derived from GenBank were obtained, including *Abies* (2), *Pinus* (2), *Picea* (3), *Pseudotsuga* (1), *Arabidopsis* (3), *Antirrhinum* (2), *Camellia* (1), *Clarkia* (2), *Cucumis* (1), *Malus* (1), *Nicotiana* (2), *Oryza* (1), *Populus* (1), *Pyrus* (1), *Salvia* (1), *Santalum* (1), *Solanum* (9), and *Vitis* (4). The TPS protein sequences of these other plants contain all the TPS subfamilies, so they were used to classify the TPS subfamilies of *P. chienii*. The accession numbers are listed in Table S16. Finally, 24 TPS protein-coding sequences from the *P. chienii* transcriptome and 38 TPS protein sequences of other plants derived from GenBank were used to construct the TPS phylogenetic tree. The filtering of the CYP450 protein-coding sequences is consistent with that of TPS. We ultimately constructed a phylogenetic tree using 112 CYP450 protein-coding sequences. The classification of the CYP450 protein-coding sequences was based on the reference sequences from a CYP450 database established by Nelson (https://drnelson.uthsc.edu/CytochromeP450.html) and the annotation results from the *P. chienii* transcriptome. The protein-coding sequences for these two gene families were individually aligned with MAFFT (version 7.037) [133] following the default settings. Phylogenetic trees were visualized and annotated using FigTree v1.4.2 [134].

## 5. Conclusions

In summary, we sequenced the full-length transcriptomes in four organs of *P. chienii* by combining the PacBio Iso-Seq and Illumina RNA-Seq technologies and obtained reference transcriptome sequences for *P. chienii*. A total of 221,101 unigenes were obtained from four organs and were functionally classified based on a similarity search across seven public databases. Then, CDSs, TFs, lncRNAs, and AS events were detected. The DEGs among the four organs and organ-specific gene expression were profiled. We identified the gene families related to biotic/abiotic factors, including the TPS, CYP450, and HSP families, which possibly participate in the regulation mechanisms underlying ecological adaptability of the four organs of *P. chienii*. The key genes involved in the phenylpropanoid biosynthesis pathway and the plant–pathogen interactions were characterized, and the expression levels of most DEGs in these two pathways were higher in the root than in the other three organs, suggesting that the root constitutes the main organ for the synthesis and accumulation of defensive compound and has a stronger ability to respond to stress and adaptability. These transcriptome data comprise the first comprehensive gene expression profiles across different organs of *P. chienii*. This study not only provides a practical guide for the transcriptomic analysis of species lacking genomic information but will also facilitate further studies on functional genomics, adaptive evolution, and phylogeny and lay a foundation for the development of conservation strategies for this endangered conifer.

## Figures and Tables

**Table 1:** Statistics of annotations of the full-length transcripts from four organs of *P. chienii* with seven databases.

Database	Root	Stem	Leaf	Strobilus
Nr	38,641	45,531	46,770	53,725
Swiss-Prot	34,034	41,986	36,644	50,386
KEGG	37,452	43,929	46,196	51,357
KOG	26,123	31,452	26,955	38,723
GO	27,425	34,770	30,132	40,467
Nt	23,665	23,851	32,373	28,089
Pfam	27,425	34,770	30,132	40,467
At least one database	40,166 (89.46%)	49,593 (85.38%)	47,697 (94.48%)	58,654 (86.72%)
All databases	13,610 (30.31%)	13,811 (23.78%)	15,489 (30.68%)	16,641 (24.60%)

## Supplementary Materials

The following are available online at https://www.mdpi.com/1422-0067/21/12/4305/s1, Figure S1. Overview of the data processing pipeline; Figure S2. The results of the transcriptome integrity assessment based on BUSCO using an Embryophyta (ODB9) core gene dataset. The number of Embryophyta gene sets used in this evaluation was 1440; Figure S3. Gene Ontology (GO) classification analysis of unigenes; Figure S4. The Kyoto Encyclopedia of Genes and Genomes (KEGG) pathway classification statistics of the unigenes; Figure S5. The features of long non-coding RNAs (lncRNAs) in four organs. (a) Length distribution of the identified lncRNAs in four organs; (b) A heatmap of identified lncRNAs; Figure S6. Analysis of gene expression in four organs of *P. chienii*. (a) The FPKM interval distribution in four organs; (b) A boxplot of expression levels in four organs; (c) A venn diagram of the number of unigenes expressed in four organs; Figure S7. The Kyoto Encyclopedia of Genes and Genomes (KEGG) pathway enrichment analysis of 16,562 differentially expressed genes (DEGs); Figure S8. Top 10 most abundant cytochrome P450s (CYP450s) in four organs of *P. chienii*; Figure S9. A heatmap of the identified heat shock protein (HSP) unigenes. A scale indicates the color assigned to log_10_(FPKM+1). Red indicates high expression, and blue indicates low expression. Table S1. Summary for the transcriptome data of *P. chienii* using PacBio Iso-Seq; Table S2. Statistics of the reference transcript sequences of *P. chienii* using PacBio Iso-Seq; Table S3. Summary of the reads sequenced using Illumina RNA-Seq; Table S4. Statistics of the unigenes sequenced using Illumina RNA-Seq; Table S5. Comparison of the unigene length distribution for different sequencing platforms; Table S6. Gene Ontology (GO) annotation of the unigenes in four organs of *P. chienii*; Table S7. Unigene functional classification by Kyoto Encyclopedia of Genes and Genomes (KEGG) in four organs of *P. chienii*; Table S8. Coding sequences (CDSs) identification for four organs of *P. chienii* from PacBio Iso-Seq; Table S9. Identification of transcription factors (TFs) in four organs of *P. chienii*; Table S10. The mapping between the Illumina reads of each sample and the reference transcript sequences generated by PacBio Iso-Seq; Table S11. Differentially expressed genes (DEGs) from the comparisons of the root, stem, leaf, and strobilus; Table S12. The number of upregulated and downregulated differentially expressed genes (DEGs) for leaf vs. strobilus, leaf vs. root, leaf vs. stem, strobilus vs. root, strobilus vs. stem, and stem vs. root; Table S13. Gene Ontology (GO) enrichment analysis of differentially expressed genes (DEGs) from paired combinations of root, stem, leaf, and strobilus; Table S14. Kyoto Encyclopedia of Genes and Genomes (KEGG) pathway enrichment analysis of differentially expressed genes (DEGs) from paired combinations of root, stem, leaf, and strobilus; Table S15. Statistics of the gene families related to biotic/abiotic factors for four organs of *P. chienii*; Table S16. Statistics of the terpene synthase (TPS) gene family; Table S17. Statistics of the cytochrome P450 (CYP450) gene family; Table S18. Statistics of the heat shock protein (HSP) gene family; Table S19. Statistics of the unigenes in the phenylpropanoid biosynthesis pathway; Table S20. Statistics of the differentially expressed genes (DEGs) in the phenylpropanoid biosynthesis pathway; Table S21. Statistics of the unigenes in the plant–pathogen interactions; Table S22. Statistics of the differentially expressed genes (DEGs) in the plant–pathogen interactions.
