# Supplementary material for: Characterization and Analysis of the Full-Length Transcriptomes of Multiple Organs in Pseudotaxus chienii (W.C.Cheng) W.C.Cheng
Source: Int J Mol Sci. 2020 Jun 17;21(12):4305. doi: 10.3390/ijms21124305 (PMC7352595; doi:10.3390/ijms21124305)
Supplement: Supplementary file 1 [file ijms-21-04305-s001.zip › Supplementary Files/Supplementary Files.docx]

**Supplementary Files**

Characterization and Analysis of the Full-Length Transcriptomes of Multiple Organs in *Pseudotaxus chienii* (W.C.Cheng) W.C.Cheng

Li Liu ^1^, Zhen Wang ^1^, Yingjuan Su ^1,2,^* and Ting Wang ^3,^*

^1^ School of Life Sciences, Sun Yat-sen University, Guangzhou 510275, China; liuli11307@163.com (L.L.); zhenwangzhenwang@163.com (Z.W.)

^2^ Research Institute of Sun Yat-sen University in Shenzhen, Shenzhen 518057, China

^3^ College of Life Sciences, South China Agricultural University, Guangzhou 510642, China

***** Correspondence: suyj@mail.sysu.edu.cn (Y.S.); tingwang@scau.edu.cn (T.W.); Tel.: +86-020-84111939 (Y.S.); +86-020-85280185 (T.W.)

**Table S1.** Summary for the transcriptome data of *P. chienii* using PacBio Iso-Seq.

| **Sample** | **Subreads** | | | **CCSs** | | | | **Consensus isoforms number** | **Unigenes** | | | |
| --- | --- | --- | --- | --- | --- | --- | --- | --- | --- | --- | --- | --- |
|  | **Subreads bases (G)** | **Subreads number** | **Mean subreads length (bp)** | **CCSs number** | **FLNC reads** | **Mean FLNC reads length (bp)** | **FLNC reads N50 (bp)** |  | **Total nucleotide bases (bp)** | **Unigenes number** | **Mean unigenes length (bp)** | **Unigenes N50 (bp)** |
| root | 8.21 | 3,589,223 | 2289 | 223,184 | 174,922 | 2585 | 2744 | 80,434 | 125,875,275 | 44,896 | 2804 | 3079 |
| stem | 8.35 | 4,242,683 | 1968 | 212,703 | 175,321 | 2514 | 2788 | 92,515 | 147,473,828 | 58,082 | 2539 | 2965 |
| leaf | 11.42 | 4,905,002 | 2328 | 325,323 | 270,445 | 2703 | 3015 | 142,159 | 146,148,395 | 50,485 | 2895 | 3232 |
| strobilus | 11.22 | 5,427,724 | 2068 | 331,286 | 269,571 | 2347 | 2543 | 126,538 | 171,096,692 | 67,638 | 2530 | 2767 |
| Total/mean |  |  |  | 1,092,496 | 890,259 | 2537 | 2773 |  |  | 221,101 | 2692 | 3010.75 |

CCS, circular consensus sequence; FLNC, full-length non-chimeric.

**Table S2.** Statistics of the reference transcript sequences of *P. chienii* using PacBio Iso-Seq.

| **Total nucleotide bases (bp)** | **Total number** | **Mean length (bp)** | **Min length (bp)** | **Max length (bp)** | **N50 (bp)** | **N90 (bp)** |
| --- | --- | --- | --- | --- | --- | --- |
| 529,056,695 | 197,174 | 2683 | 160 | 14,887 | 3024 | 1877 |

**Table S3.** Summary of the reads sequenced using Illumina RNA-Seq.

| **Sample** | **Raw reads** | **Clean reads** | **Clean bases (G)** | **Error (%)** | **Q20 (%)** | **Q30 (%)** | **GC content (%)** |
| --- | --- | --- | --- | --- | --- | --- | --- |
| root | 52,633,598 | 51,073,864 | 7.66 | 0.03 | 97.16 | 92.16 | 44.25 |
| stem | 57,112,536 | 56,419,624 | 8.46 | 0.03 | 98.03 | 94.03 | 46.26 |
| leaf | 62,840,720 | 61,110,258 | 9.17 | 0.03 | 96.9 | 91.55 | 45.57 |
| strobilus | 42,617,842 | 41,448,574 | 6.22 | 0.03 | 97.28 | 92.34 | 45.23 |

Q20, the percentage of bases with a Phred value ≥ 20; Q30, the percentage of bases with a Phred value ≥ 30.

**Table S4.** Statistics of the unigenes sequenced using Illumina RNA-Seq.

| **Sample** | **Min length (bp)** | **Mean length (bp)** | **Max length (bp)** | **N50 (bp)** | **Total nucleotide bases (bp)** | **Total number** |
| --- | --- | --- | --- | --- | --- | --- |
| root | 201 | 1328 | 13,579 | 1843 | 87,790,725 | 66,126 |
| stem | 201 | 1270 | 12,558 | 1728 | 101,369,156 | 79,842 |
| leaf | 201 | 1421 | 11,562 | 1931 | 74,169,328 | 52,207 |
| strobilus | 201 | 1262 | 12,275 | 1754 | 76,205,289 | 60,391 |
| Mean | 201 | 1320.25 |  | 1814 |  |  |

**Table S5.** Comparison of the unigene length distribution for different sequencing platforms.

| **length distribution (bp)** | **PacBio** **Iso-seq** | | **Illumina RNA-seq** |
| --- | --- | --- | --- |
|  | **Consensus isoforms** | **Unigenes** | **Unigenes** |
| <500 | 11,923 (2.70%) | 4897 (2.21%) | 47,969 (18.55%) |
| 500-1000 | 21,655 (4.90%) | 10,578 (4.78%) | 81,996 (31.71%) |
| 1000-2000 | 89,407 (20.24%) | 43,121 (19.50%) | 78,714 (30.44%) |
| 2000-3000 | 166,650 (37.73%) | 87,033 (39.36%) | 32,014 (12.38%) |
| 3000-4000 | 117,666 (26.64%) | 52,048 (23.54%) | 11,347 (4.39%) |
| 4000-5000 | 24,019 (5.44%) | 14,983 (6.78%) | 4194 (1.62%) |
| >5000 | 10,326 (2.34%) | 8441 (3.82%) | 2332 (0.90%) |

**Table S8.** Coding sequences (CDSs) identification for four organs of *P. chienii* from PacBio Iso-Seq.

|  | **root** | **stem** | **leaf** | **strobilus** |
| --- | --- | --- | --- | --- |
| CDS | 45,020 | 58,539 | 50,842 | 68,851 |
| Completed CDS | 26,881 | 32,398 | 29,235 | 39,633 |
| 3′ partial | 748 | 1236 | 1079 | 1145 |
| 5′ partial | 5923 | 7732 | 7480 | 7691 |
| Uncertain | 11,468 | 17,173 | 13,048 | 20,382 |
| 5‘UTR | 37,204 | 45,390 | 42,602 | 55,482 |
| 3’UTR | 43,643 | 55,685 | 49,110 | 66,243 |

CDS, coding sequence; UTR, untranslated region.

**Table S10.** The mapping between the Illumina reads of each sample and the reference transcript sequences generated by PacBio Iso-Seq.

| **Sample** | **Total reads** | **Total mapped** |
| --- | --- | --- |
| root | 51,073,864 | 43,867,388 (85.89%) |
| stem | 56,419,624 | 47,373,258 (83.97%) |
| leaf | 61,110,258 | 54,728,764 (89.56%) |
| strobilus | 41,448,574 | 35,154,778 (84.82%) |

**Table S12.** The number of upregulated and downregulated differentially expressed genes (DEGs) for leaf vs. strobilus, leaf vs. root, leaf vs. stem, strobilus vs. root, strobilus vs. stem, and stem vs. root.

| **DEGs set name** | **Number of**  **DEGs** | **Upregulated**  **DEGs** | **Downregulated**  **DEGs** | **Annotated** |
| --- | --- | --- | --- | --- |
| leaf vs. strobilus | 7119 | 3185 | 3934 | 6921 |
| leaf vs. root | 9660 | 4092 | 5568 | 9403 |
| leaf vs. stem | 6742 | 3133 | 3609 | 6500 |
| strobilus vs. root | 6985 | 3216 | 3769 | 6750 |
| strobilus vs. stem | 6881 | 3472 | 3409 | 6595 |
| stem vs. root | 6321 | 2898 | 3423 | 6087 |

**Table S15.** Statistics of the gene families related to biotic/abiotic factors for four organs of *P. chienii*.

| **Gene families** | | **root** | **stem** | **leaf** | **strobilus** |
| --- | --- | --- | --- | --- | --- |
| terpene synthase (TPS) | | 237 | 67 | 94 | 104 |
| cytochrome P450 (CYP450) | | 734 | 362 | 409 | 487 |
| Heat shock protein (HSP) | HSP100 | 4 | 12 | 0 | 23 |
|  | HSP90 | 45 | 76 | 39 | 88 |
|  | HSP70 | 165 | 233 | 123 | 330 |
|  | HSP60 | 2 | 6 | 1 | 14 |
|  | small HSP (sHSP)/HSP20 | 7 | 38 | 2 | 9 |


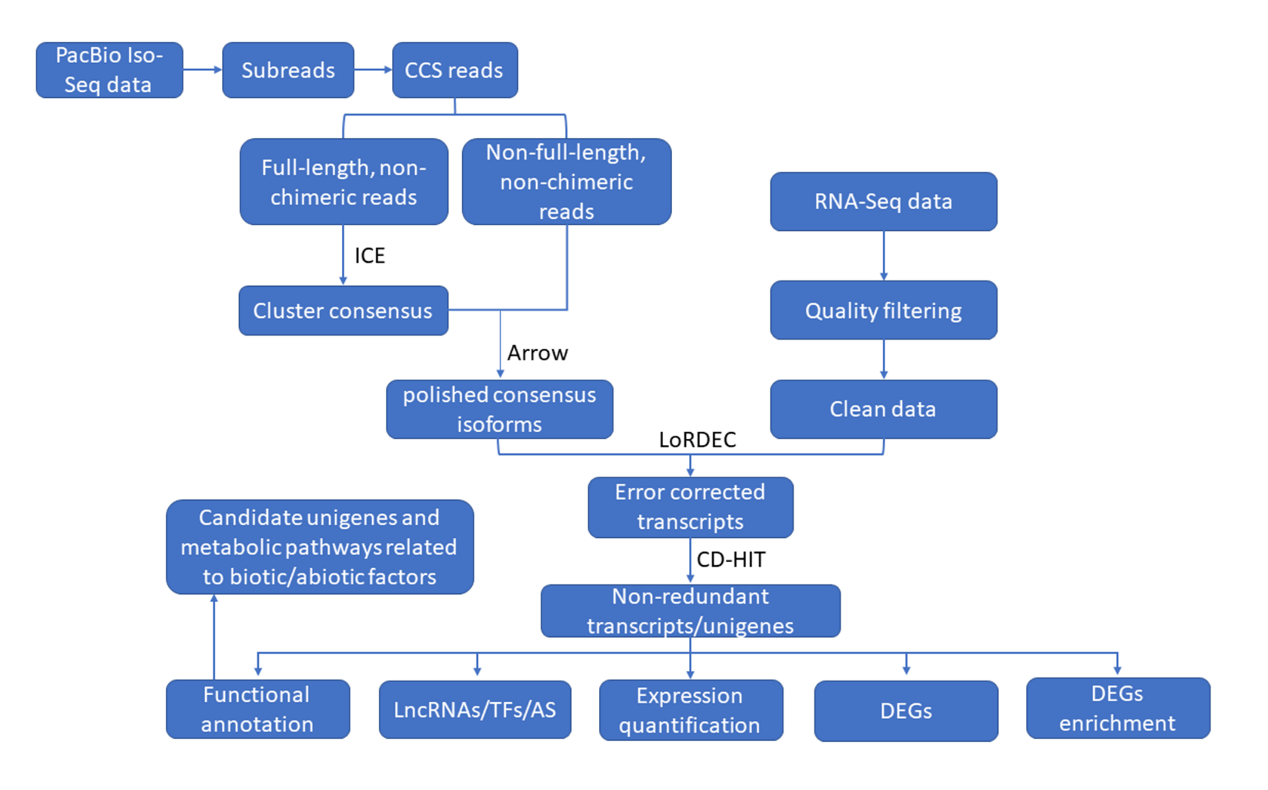


Figure S1. Overview of the data processing pipeline.


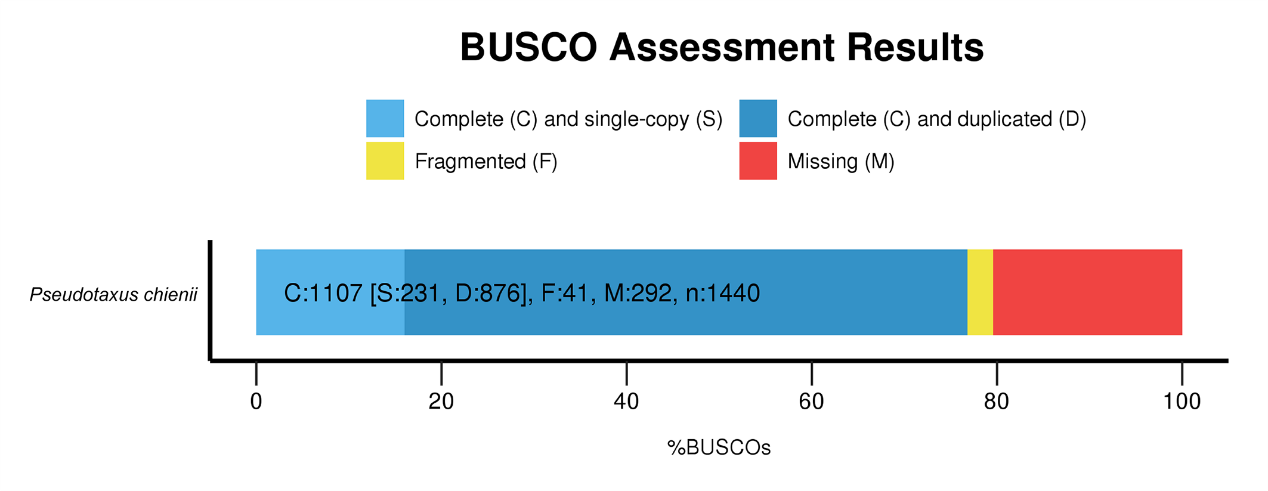


Figure S2. The results of the transcriptome integrity assessment based on BUSCO using an Embryophyta (ODB9) core gene dataset. The number of Embryophyta gene sets used in this evaluation was 1440.


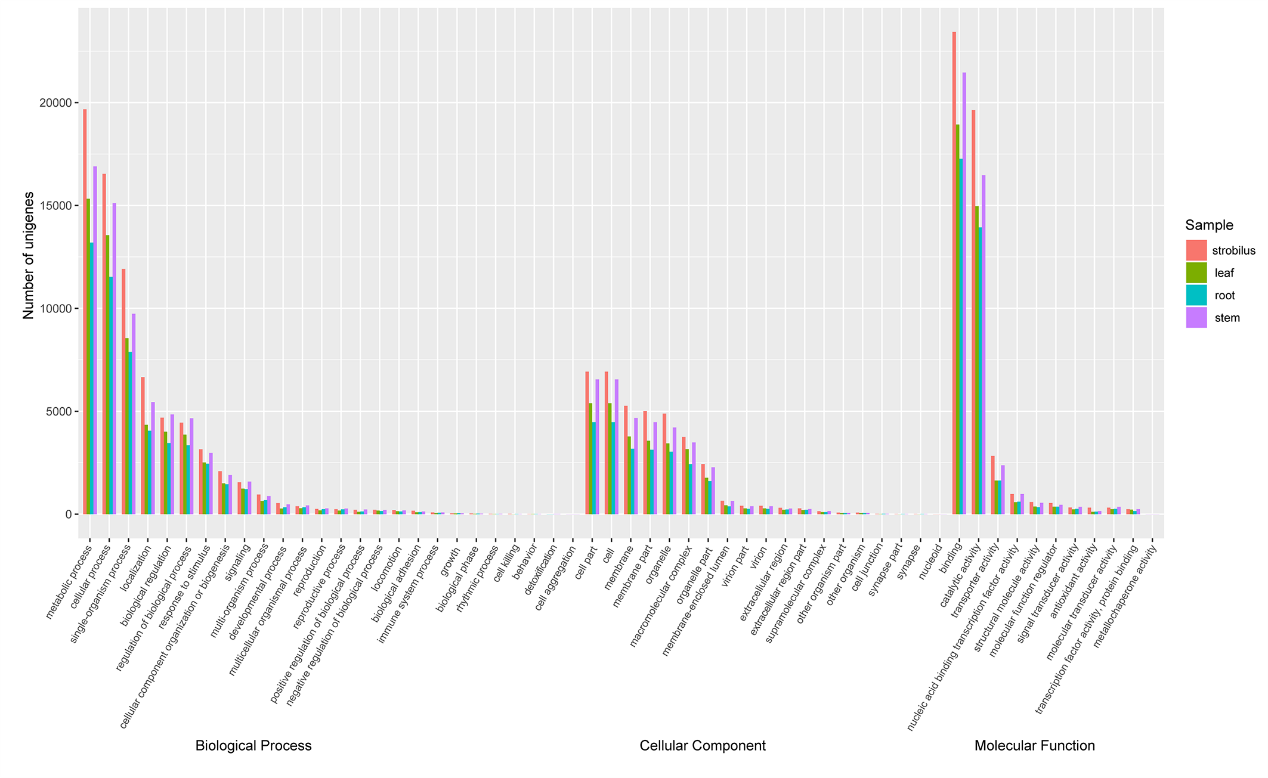


Figure S3. Gene Ontology (GO) classification analysis of unigenes.


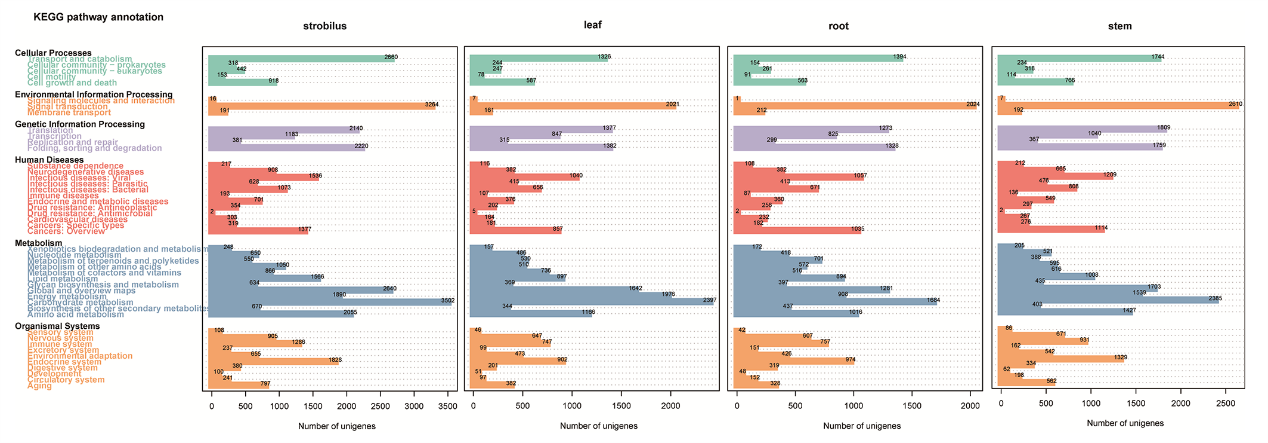


Figure S4. The Kyoto Encyclopedia of Genes and Genomes (KEGG) pathway classification statistics of the unigenes.


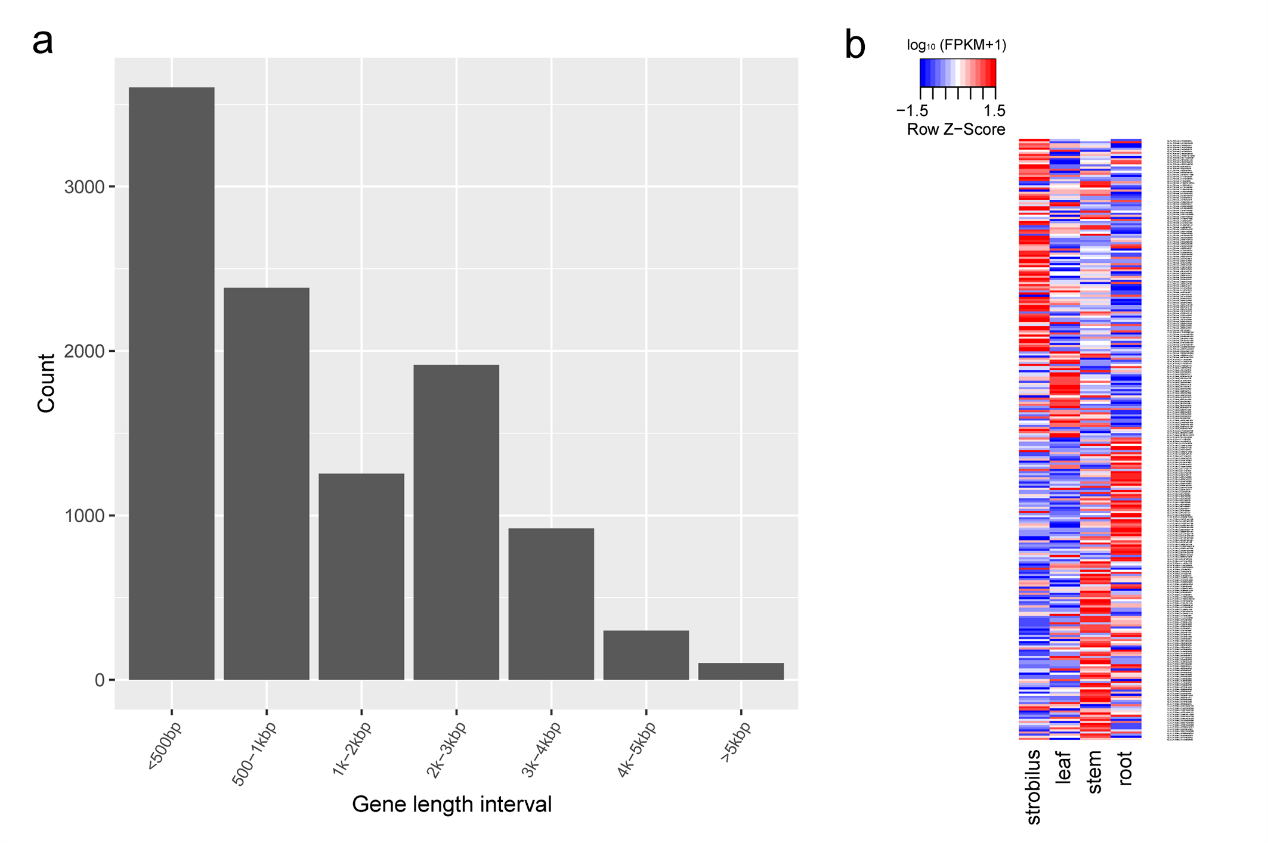


Figure S5. The features of long non-coding RNAs (lncRNAs) in four organs. (a) Length distribution of the identified lncRNAs in four organs; (b) A heatmap of identified lncRNAs.


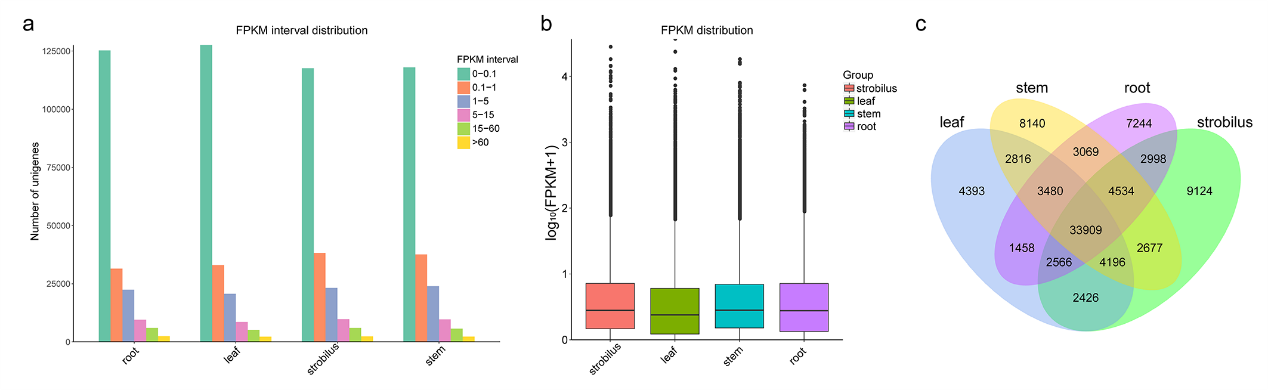


Figure S6. Analysis of gene expression in four organs of *P. chienii*. (a) The FPKM interval distribution in four organs; (b) A boxplot of expression levels in four organs; (c) A venn diagram of the number of unigenes expressed in four organs.


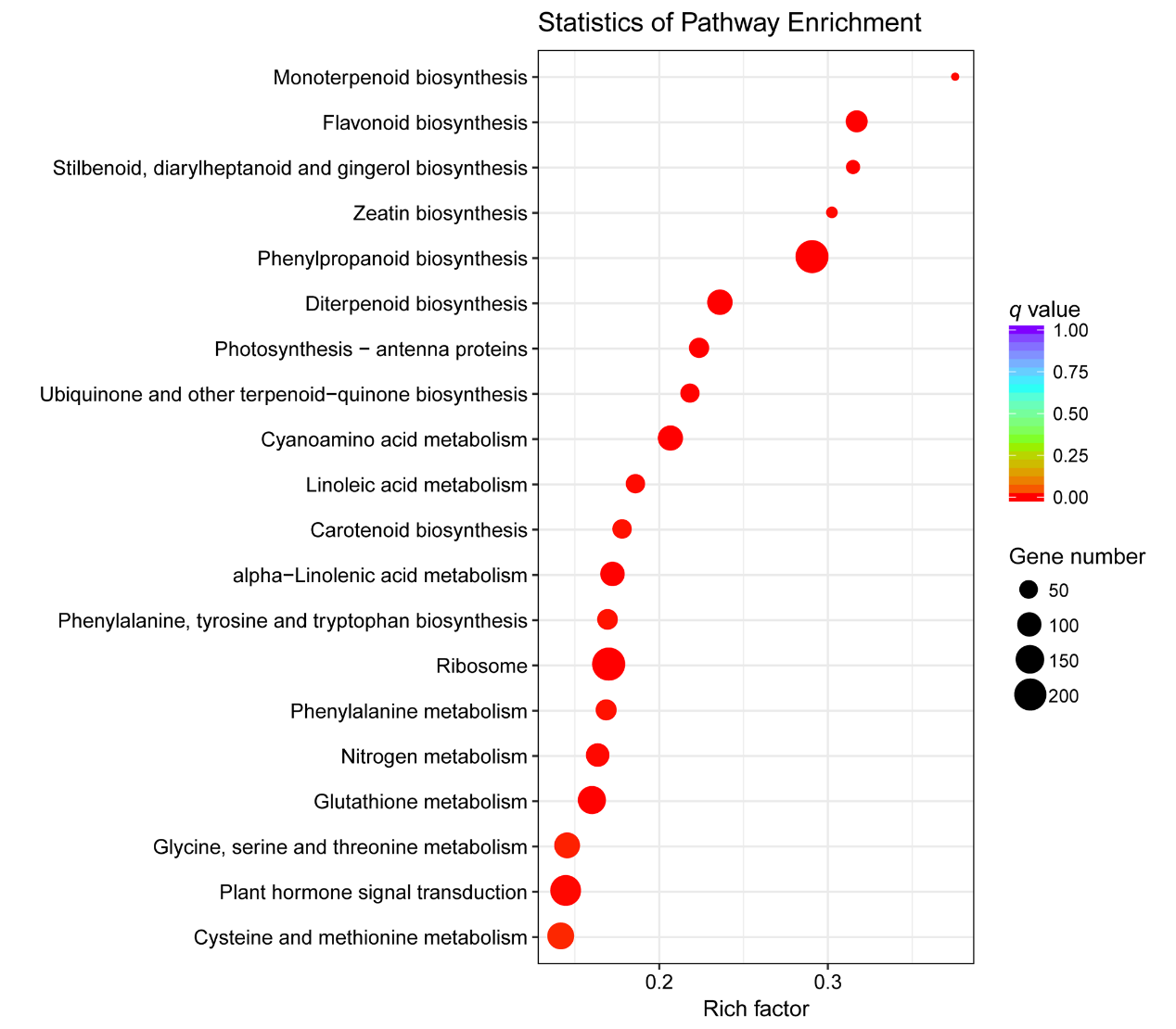


Figure S7. The Kyoto Encyclopedia of Genes and Genomes (KEGG) pathway enrichment analysis of 16,562 differentially expressed genes (DEGs).


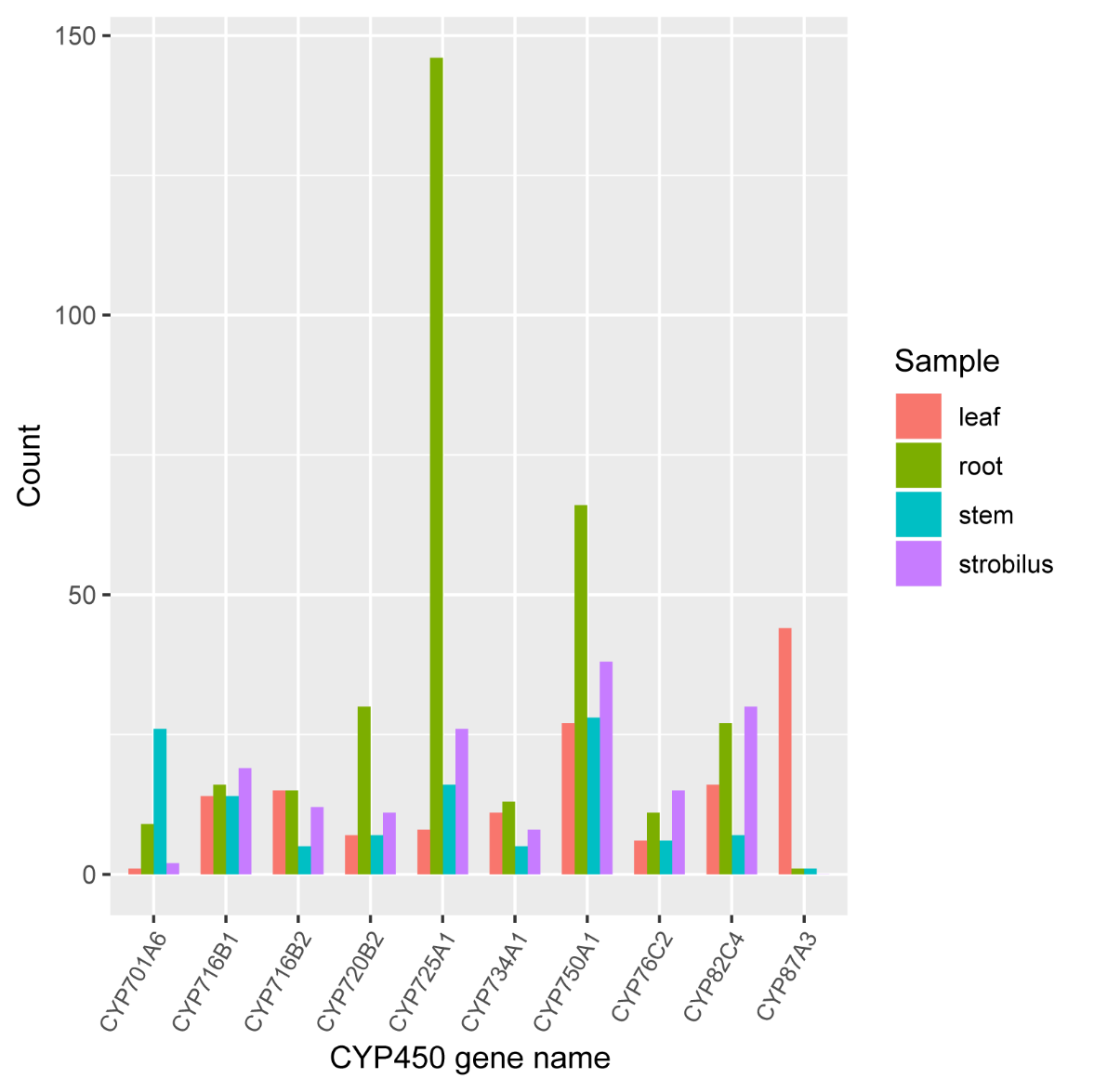


Figure S8. Top 10 most abundant cytochrome P450s (CYP450s) in four organs of *P. chienii*.


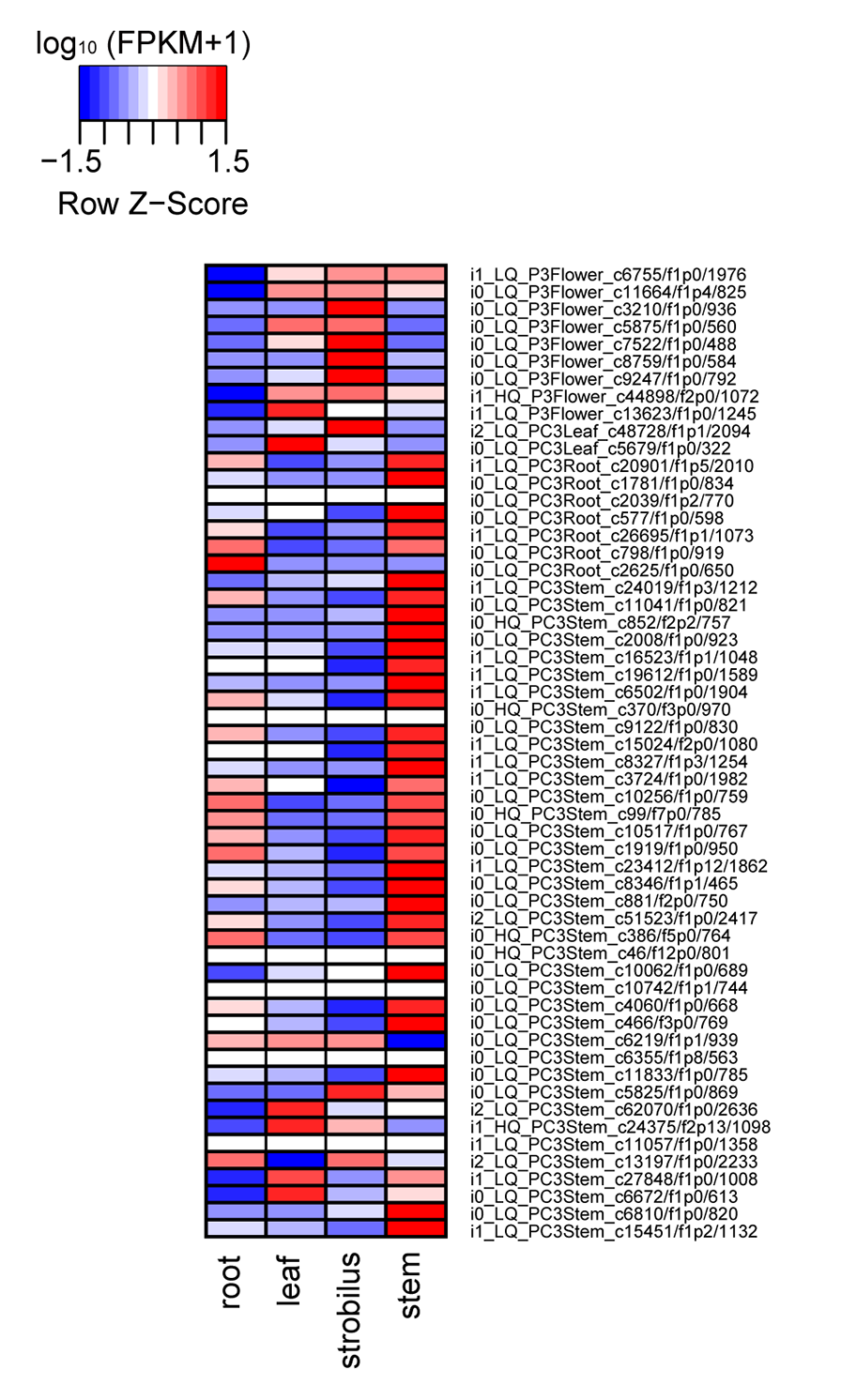


Figure S9. A heatmap of the identified heat shock protein (HSP) unigenes. A scale indicates the color assigned to log_10_(FPKM+1). Red indicates high expression, and blue indicates low expression.
